# Host biological factors and geographic locality influence predictors of parasite communities in sympatric sparid fishes off the southern Italian coast

**DOI:** 10.1038/s41598-020-69628-1

**Published:** 2020-08-06

**Authors:** Mario Santoro, Doriana Iaccarino, Bruno Bellisario

**Affiliations:** 1 Department of Integrative Marine Ecology, Stazione Zoologica Anton Dohrn, Villa Comunale 1, 80121 Naples, Italy; 2 Department of Animal Health, Istituto Zooprofilattico Sperimentale del Mezzogiorno, Via Salute 9, 80055 Portici, Italy; 3 Centre of Molecular and Environmental Biology, University of Minho, Campus de Gualtar, 4710-057 Braga, Portugal; 4 Institute of Science and Innovation for Bio-Sustainability, University of Minho, Campus de Gualtar, 4710-057 Braga, Portugal; 5 Department of Ecological and Biological Sciences, Largo Dell’Università Snc, University of Viterbo, 01100 Viterbo, Italy

**Keywords:** Ecology, Zoology

## Abstract

Host biological factors and habitat influence the faunal assemblages and biodiversity worldwide, including parasite communities of vertebrate and invertebrate hosts. The ecological relationship between hosts and parasites can be mediated by interaction of host’s biological factors, as their physiological condition, diet and size, with the environmental components, somehow influencing the features of parasite infection in host populations. Here, we used boosted regression tree models to study the parasite communities of two sympatric sparid fishes, the salema *Sarpa salpa* and the white seabream *Diplodus sargus,* to investigate the role of specific host’s traits in two contiguous coastal areas along the southern-western Tyrrhenian coast of Italy characterized by different degree of deterioration. Results showed that overall and across all parasite groups (ecto-, endo- and ecto- plus endo-parasites), sampling localities were the most important predictors of abundance, species richness, and diversity for salema. Moreover, seasonality was the main predictor of endo-parasite abundance, while size-related factors explained most of the variation in species richness and diversity. In the white seabream, size-related factors and reproductive cycle-related factors were the most important predictors for the overall parasite abundance and parasite richness, respectively. Our findings suggest that the parasite community of salema and white seabream responded differently to specific biological factors, highlighting how the environmental conditions under which they live may exert a strong influence on the parasite communities of each host fish.

## Introduction

The parasite community of marine hosts is influenced by the interactions that occur between hosts, parasites, and their environment. Biological (or biotic) factors affecting the parasite community structure in a given host and locality may be altered by several stressors of both natural and anthropogenic nature^1–5^. High impact due to human activities can cause changes to the assemblages and biodiversity of coastal fauna, including parasite community of vertebrate and invertebrate hosts^1–4,6,7^. Metazoan parasites are considered to be sensitive to environmental stress and potential bio-indicators of water quality and environment stability. Their community’s descriptors have been considered as effective indicators that reflect habitat alterations and have been used to evaluate the environment health status and anthropogenic impact in coastal habitats^2–4,8–13^.

The Gulf of Naples and Gulf of Salerno are both located along the Tyrrhenian coast of southern Italy. The first area is a semi-closed basin affected by significant degradations, especially along the coast. The strong pressure due to high population density and agricultural and industrial activities has resulted in the general deterioration of the marine environment by urban and industrial sewages and river discharges^14–16^. In particular, on its south-eastern part, the basin is strongly affected by the pulsing runoff of the Sarno River, which is considered the most polluted European river featuring a mix of sewage and untreated agricultural and industrial wastes and chemicals^14,15^. Its sediments cause eutrophication that in turn can enhance changes in the composition of coastal fauna and its food web ^14,15^. The communication with the southern contiguous basin of the Gulf of Salerno, which in comparison with the Gulf of Naples shows a reduced anthropogenic pressure^14–16^, is through the passage between the Island of Capri and the Sorrento Peninsula.

The salema *Sarpa salpa* and the white seabream *Diplodus sargus* (Sparidae) are demersal and sympatric species that inhabit coastal rocky reef areas and *Posidonia oceanica* meadows. Both species are among the most common and abundant sparid fishes in shallow waters of the Mediterranean Sea, so that they could easily be used as sentinels of environment stability. Moreover, because they show different feeding ecology, the simultaneous study of their parasite communities provides the opportunity to obtain information from different trophic levels. Salema is largely herbivorous grazing on aquatic plants^17^, in contrast, invertebrates and different algal species compose the diet of the white seabream^18^. To our knowledge, no study has been conducted on the whole metazoan parasite community of salema and white seabream from the Tyrrhenian Sea or in other sparid fishes from off southern Italian coast.

Herein, we use boosted regression tree models (BRTm) to investigate the influence of biological factors and geographic localities on the descriptors of parasite communities in salema and white seabream in two contiguous basins characterized by different ecosystems and degrees of alteration along the southern-western Tyrrhenian coast of Italy. Our results show that biological factors and geographic locality affect the parasite community of the two fishes, supporting the idea that the deterioration of ecosystems may play an important role on fish hosts that, in turn, could be used as biological indicators.

## Methods

### Study area

**Figure 1:**
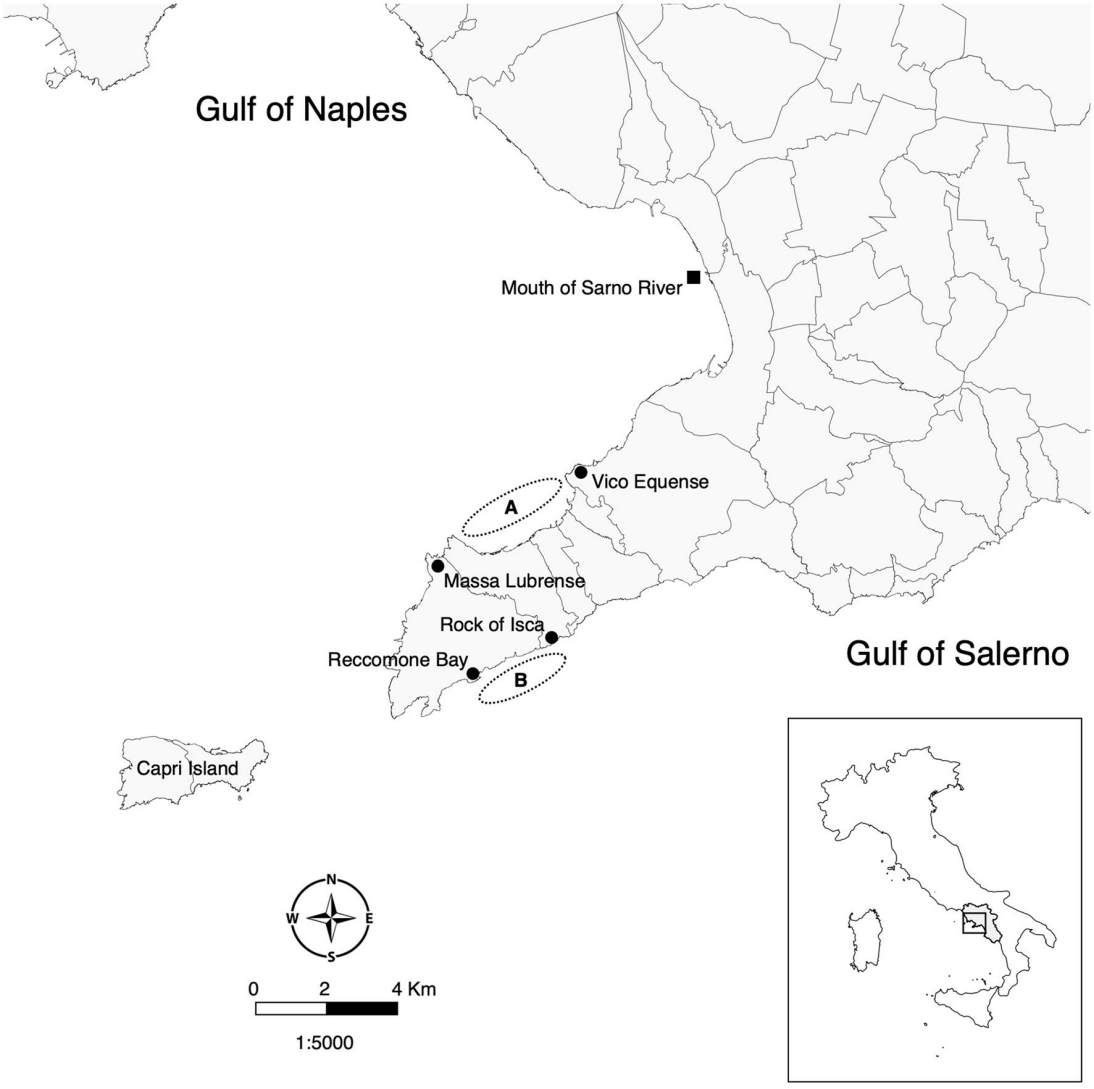
Sampling areas from the Gulf of Naples (A) and Gulf of Salerno (B). Black dots represent the areas within which fishes have been sampled and the black square corresponds to the mouth of Sarno River.

For comparative purposes, two areas known to have different degree of deteriorations due to human impact have been selected for this study: the first area is located along the coastline between Vico Equense and Massa Lubrense in the Gulf of Naples, and the second between Recommone Bay and the Rock of Isca in the Gulf of Salerno (Fig. 1). The two areas will further be referred to as GN (Gulf of Naples) and GS (Gulf of Salerno), respectively. GN and GS are located along the Tyrrhenian Sea, Campania region, (southern Italy), separated by the Sorrento Peninsula. GN is strongly influenced by heavy pollution due to anthropogenic impact^14–16^, while GS is located just outside the marine protected area of Punta Campanella, where the anthropogenic pressure is known to be strongly reduced^14^.

### Collection and fish examination

All the fish specimens were collected during normal fishery procedures with no additional experimental catches being performed. According to Italian law DL16/92 and European directive 2010/63/EU, this study did not require a specific permit. Procedures for this study were performed in accordance with the guide for the care and use of animals by the Italian Ministry of Health.

Between February 2017 and October 2018, a total of 242 individual fish of two species of Sparidae were collected by net at benthic depths ranging from 15 to 30 m. Sampling included 64 and 56 specimens of white seabream from GN and GS, respectively and 60 and 62 specimens of salema from GN and GS, respectively. However, due to extreme weather events in the GS, it was not possible to obtain white seabream individuals in winter 2017. For this latter reason, the timing factors including season (winter, spring, summer and autumn) and year have been not considered in the statistical analysis for the white seabream.

Fish were weighed to the nearest 0.1 g and measured (fork length-FL) to nearest 0.1 cm; sex was determined before parasitological study by gonadal examination^19^. A macroscopic gonadal maturity score (GMS) was recorded to investigate the phase of the reproductive cycle (1 = inactive; 2 = developing; 3 = ripe; 4 = post spawning)^19^. Body condition index (BCI, whole weight/fork length^3^) was calculated as described by Le Cren^20^. The gonadosomatic (GSI, gonad weight/host eviscerated weight × 100) and hepatosomatic indices (HSI, liver weight/host eviscerated weight × 100) were calculated as suggested by Mouine et al.^19^.

The skin, musculature, gills, mouth cavity, digestive tract, liver, heart, gonads, visceral cavity and mesenteries of each fresh individual fish were examined under a dissecting microscope for parasites. For each organ, ecto- and endo-parasites were collected, counted, washed in physiological saline solution, and preserved in 70% ethanol^21^. For identification, crustaceans and nematodes were clarified in 20% potassium hydroxide and Amman’s lactophenol respectively, and then returned to 70% ethanol, trematodes were stained with Mayer’s acid carmine and mounted in Canada balsam. Parasites were studied by a compound microscope.

### Descriptors of parasite community

A component community comprises all the species of parasites recovered from a sample of a particular host species, while infracommunity refers to the assemblage of parasites in one host individual. Prevalence was defined as the number of hosts infected with 1 or more individuals of a particular parasite species; parasite species with prevalence higher than 10% in any of the host samples will further be referred to as common. Abundance was measured as the number of individuals of a particular parasite in/on a single host regardless of whether or not the host is infected; intensity was the number of individuals of a particular parasite species in a single infected host^22^.

The mean total abundance, species richness and the Simpson and Shannon–Wiener indices of diversity were used as overall descriptors of infracommunities for each host species examined. Mean total abundance was measured as the mean number of individuals of all parasite species, and species richness the number of parasite species harboured by each individual fish. We used both diversity indices because Simpson's diversity index is able to detect differences in abundant species, whilst the Shannon–Wiener diversity index detects differences in rare species^23^. Descriptors of community were compared between hosts using the Mann–Whitney U- test.

Parasite species by host specificity were classified as ‘‘specialists’’, defined narrowly as having the bulk of reproducing adults found only in a single host species or having been reported from a single host species and ‘‘generalists’’ when reported from a variety of related host species.

### Statistical analysis

We used BRTm to assess the influence of host biological factors (sex, weight, FL, GMS, BCI, GSI, and HSI), timing (sampling season and year, available only for salema) and sampling areas (which we considered as proxies of different abiotic conditions) on parasites abundance, species richness (including zeros^22^), and diversity, measured by Shannon and Simpson indices^23^. BRT models are characterized by a great flexibility in model evaluation, allowing for non-linear responses, collinear predictors, and variable interactions^24^. Since sampling in salema took a longer period of time encompassing different seasons and years, we included those variables into the BRTm. To avoid biases in model fitting due to possible interactions between different predictors (e.g. sampling year and season in salema), we re-ran BRTm for salema by excluding those terms. Comparative analyses were conducted on gastro-intestinal plus liver parasites (endo-parasites), and external parasites (ecto-parasites; from gills and skin) separately, and then on all parasites (endo- plus ecto-parasites). Parasite descriptors (total abundance, species richness, Shannon and Simpson indices) were subdivided in total, endo- and ecto-parasites, and models were trained on all groups by means of the ‘*gbm.step*’ function in the ‘*dismo’* package of R^25^.

Models were trained by using common values of learning rate, step size and bag fraction (i.e. the proportion of observations used in selecting variables^24^), allowing for interactions among covariates. To avoid overfitting due to the limited amount of data (n_Sarpa_ = 90, n_Diplo_ = 102), the optimal number of trees was chosen by means of a cross-validation procedure (max trees = 10,000). The relative importance of each predictor was determined by measuring the increase in model fitting after accounting for a given predictor, scaled in a 0–100% scale where larger values correspond to larger (relative) influence in model fitting^26^. The relative influence of each predictor on the abundance/richness and diversity indices was measured by means of partial dependence plots visualizing the relationship between predictor and the fitted function. Model accuracy was examined using the Spearman’s rank correlation values between fitted and observed abundance, richness and diversity values for each parasite group. The strength of interaction effects in BRT models was measured by means of the H-statistic, ranging between 0 and 1, with higher values corresponding to larger interaction effects^24^.

## Results

### Host and parasite data

Biological data (including sex, weight, BCI, GMS, GSI, and HIS) of salema and white seabream individuals according to sampling localities are reported in Table 1. A total of 3,861 individual parasites belonging to 20 taxa (11 in white seabream and 10 in salema) were identified in two host species. Only larval forms of the isopod *Gnathia* sp. were found in both host species. Basic parameters of infection for each parasite taxon from both localities are presented in Table 2. All ecto-parasites were obtained from the gills, and *Gnathia* sp. from the skin also, while endo-parasites were obtained from the intestine, except for larvae of *Hysterothylacium* sp. collected from the liver. The clearly predominant group of parasites with respect to species diversity was the Digenea (8 species) followed by Monogenea (4 species). The other groups were represented by fewer species: Copepoda (3 species), Hirudinidae and Nematoda (2 species each), and Isopoda (1 species). The local parasite fauna showed a low representation of larval parasite stages (2 species: one nematode and one isopod). Only five parasite species in white seabream and four species in salema were present from both localities (Table 2).

**Table 1:** Biological data of salema *Sarpa salpa* and white seabream *Diplodus sargus* according to sampling localities: Weight (g); FL, fork length (cm); BCI, body condition index; GMS, gonadal maturity score; GSI, gonadosomatic index; HSI, hepatosomatic index. Data are presented as mean (± SD). Sex is presented as number of males (m), females (f), and hermaphrodites (h) in the sampling.

	Gulf of Naples	Gulf of Salerno
*Sarpa salpa*		
Sex	7 m/36 f/3 h	9 m/31 f/4 h
Weight	171.2 ± 52.4	183.4 ± 49.7
FL	20.2 ± 1.7	20.8 ± 1.8
BCI	0.02 ± 0.002	0.02 ± 0.002
GMS	2.2 ± 0.9	2.04 ± 0.7
GSI	0.2 ± 0.7	0.42 ± 1.1
HSI	1.8 ± 0.5	1.63 ± 1
*Diplodus sargus*		
Sex	25 m/36 f/1 h	18 m/20 f/1 h
Weight	251.3 ± 117.5	210.1 ± 165.7
FL	20.6 ± 2.6	19.5 ± 4
BCI	0.027 ± 0.005	0.024 ± 0.002
GMS	2.9 ± 0.5	2.52 ± 0.7
GSI	3.5 ± 2	3 ± 2.8
HSI	1.2 ± 0.3	0.97 ± 0.4

**Table 2:** Prevalence (P), abundance (Ab) and intensity (In) of parasite infection in the salema *Sarpa salpa* and the white seabream *Diplodus sargus* according to the sampling localities. Sampling included 60 and 62 specimens of salema from the Gulf of Naples and Salerno, respectively, and 64 and 56 specimens of white seabream from the Gulf of Naples and Salerno, respectively.

		Gulf of Naples			Gulf of Salerno			
	Location in host	P (n/%)	Ab	In	P (n/%)	Ab	In	Total P (%)
***Sarpa salpa***								
*Hirudinidae* Whitman, 1886; sp. 1	Gill	-	-	-	1/1.6	-	-	0.8
*Gnathia* sp. Leach, 1914; larval stage	Gill, skin	1/1.6	-	-	39/62.9	2.6	4.1 (1–15)	32.7
*Clavellotis briani* Benmansour, Ben Hassine, Diebakate & Raibaut, 2001	Gill	1/1.6	-	-	6/9.6	0.1	1.5 (1–3)	5.7
*Atrispinum salpae* Parona & Perugia,1890	Gill	4/6.6	0.1	1.7 (1–2)	2/3.2	0.1	3.5 (3–4)	4.9
*Lamellodiscus confusus* Amine, Euzet & Kechemir-Issad, 2007	Gill	20/33.3	0.8	2.5 (1–7)	46/74.1	6.3	8.5 (1–59)	54.0
*Mesometra brachycoelia* Lühe, 1901	Intestine	11/18.3	2.2	12.1 (1–48)	20/32.2	2.7	8.5 (1–58)	25.4
*Mesometra orbicularis* Rudolphi, 1819	Intestine	26/43.3	2.3	5.2 (1–19)	38/61.2	4.4	7.2 (1–26)	52.4
*Elstia stossichianum* Monticelli, 1892	Intestine	5/8.3	0.4	4.8 (1–10)	14/22.5	0.9	4 (1–17)	15.5
*Robphildollfusium fractum* Rudolphi, 1819	Intestine	33/55	8.3	15.1 (1–87)	48/77.4	8.6	11.1 (1–66)	66.3
*Wardula capitellata* Rudolphi, 1819	Intestine	5/8.3	0.2	2.8 (1–7)	13/20.9	0.9	4.6 (1–11)	14.7
***Diplodus sargus***								
*Hirudinidae* Whitman, 1866*;* sp. 2	Gill	-	-	-	1/1.8	-	3	0.8
*Gnathia* sp. Leach, 1914; larva	Gill, skin	17/26.5	1.1	4.3 (1–19)	22/39.2	1.1	2.9 (1–11)	32.5
*Clavellotis sargi* Kurz, 1877	Gill	-	-	-	8/14.2	0.3	2.2 (1–7)	6.6
*Hatschekia* sp. Poche, 1902	Gill	12/18.7	0.8	4.2 (1–19)	20/35.7	6.4	17.8 (1–84)	26.6
*Chorycotyle chrysophrii* Van Beneden & Hesse, 1863	Gill	-	-	-	1/1.8		4	0.8
*Lamellodiscus ignoratus* Palombi, 1943	Gill	23/35.9	6.3	17.6 (2–60)	27/48.2	6.9	14.3 (1–81)	41.6
*Lepocreadium pegorchys* Stossich, 1901	Intestine	8/12.5	0.4	3.1 (1–7)	8/14.2	0.1	1.6 (1–2)	13.3
*Holorchis pycnoporus* Stossich, 1901	Intestine	5/7.8	0.5	7 (1–14)	-	-	-	4.1
*Cucullanus campanae* Lebre & Petter,1984	Intestine	7/10.9	0.3	2.4 (1–5)	9/16	0.4	2.4 (1–6)	13.3
*Wardula sarguicola* Bartoli & Gibson, 1989	Intestine	2/3.1	0.04	1 (1–3)	-	-	-	1.6
*Hysterothylacium* sp. Ward & Magath, 1917; larval stage	Liver	1/1.5	-	1	2/3.5	0.08	2.5 (2–3)	2.5

Out of the 1,481 parasite specimens found in white seabream*,* 567 (38.2%) were ecto-parasites and 914 (61.8%) endo-parasites. Adult parasites of white seabream were all generalist in Sparidae. Out of the 2,380 parasite specimens found in salema, 468 (19.6%) were ecto-parasites and 1,912 (80.4%) endo-parasites. Adult parasites of salema were all specialist species. Overall prevalence of infection was 71.6% and 88.5% in white seabream and salema, respectively.

In white seabream, the most prevalent and abundant species was *Lamellodiscus ignoratus* (Monogenea), while in general endo-parasites showed low prevalence and abundance (Table 2). In salema, the most prevalent and abundant species was *Robphildollfusium fractum* (Digenea) with endo-parasite prevalence ranging from 14.7 to 66.3, depending on parasite species (Table 2). Both endo-parasite communities were dominated by digeneans. Endo-parasites were all trophically transmitted helminths.

### Parasite communities

In white seabream, the number of parasite species ranged from 1 to 5, with the maximum number of species observed in a single individual host. The most frequent numbers of parasite species observed per host were one and two in 32 and 31 individual hosts, respectively.

In salema, the number of parasite species ranged from 1 to 6, with the maximum number of species observed in nine individuals from the GS. The most frequent numbers of parasite species observed per host were two and four in 32 and 22 individual hosts, while the abundance ranged from 1 to 153 and from 1 to 140 in white seabream and salema, respectively.

Descriptors of parasite infracommunities for both host species are listed in Table 3. The white seabream and salema showed significant differences in parasite richness, abundance and diversity when considering the total and endo-parasite community composition, showing higher values in salema. The ecto-parasite communities did not show differences between host species (Table 3).

**Table 3:** Average values (± SD) and range (values in square brackets) of measured parameters for total, endo- and ecto-parasites in the salema *Sarpa salpa* and white seabream *Diplodus sargus*. *U* is the Mann–Whitney statistic and *p* the significance value (in bold those with significance *p* < 0.05) of their differences between populations of different host species.

	*Sarpa salpa*		*Diplodus sargus*		*U*	*p*
Total abundance	21.933 (± 25.626)	[1–136]	9.411 (± 15.476)	[1–67]	6,428	< **0.001**
Endo-parasite abundance	15.989 (± 23.872)	[1–136]	1.058 (± 2.371)	[1–71]	7,257	< **0.001**
Ecto-parasite abundance	5.945 (± 10.364)	[1–68]	8.352 (± 14.852)	[0–14]	4,404	0.619
Total species richness	2.7 (± 1.686)	[1–6]	1.313 (± 1.168)	[1–4]	6,787	< **0.001**
Endo-parasite species richness	1.722 (± 1.391)	[1–5]	0.343 (± 0.588)	[1–2]	7,257	< **0.001**
Ecto-parasite species richness	0.978 (± 0.911)	[1–4]	0.971 (± 0.938)	[1–4]	4,653	0.863
Total Shannon index	0.689 (± 0.495)	[0.223–1.789]	0.267 (± 0.363)	[0.166–1.986]	6,791	< **0.001**
Endo-parasite Shannon index	0.459 (± 0.437)	[0.274–1.986]	0.031 (± 0.128)	[0.376–0.693]	7,257	< **0.001**
Ecto-parasite Shannon index	0.178 (± 0.281)	[0.223–1.193]	0.157 (± 0.282)	[0.154–1.115]	4,653	0.863
Total Simpson index	0.504 (± 0.282)	[0.229–0.795]	0.469 (± 0.403)	[0.237–0.8]	5,009	0.271
Endo-parasite Simpson index	0.562 (± 0.331)	[0.278–0.8]	0.736 (± 0.428)	[0.245–0.5]	3,238	< **0.001**
Ecto-parasite Simpson index	0.493 (± 0.427)	[0.219–0.787]	0.472 (± 0.441)	[0.268–0.5]	4,734	0.696

BRTm were able to accurately predict the abundance, richness and diversity of all parasite groups (i.e. total, ecto- and endo-parasites, *p* < 0.001) having, on average, a higher prediction accuracy in salema ($\overline{\rho } > 0.6$) than in white seabream ($\overline{\rho } < 0.5$) and, for both species, a lower accuracy in identifying the most important predictors for endo-parasites communities (salema, $\overline{\rho } = 0.56$; white seabream, $\overline{\rho } = 0.466$).

**Figure 2:**
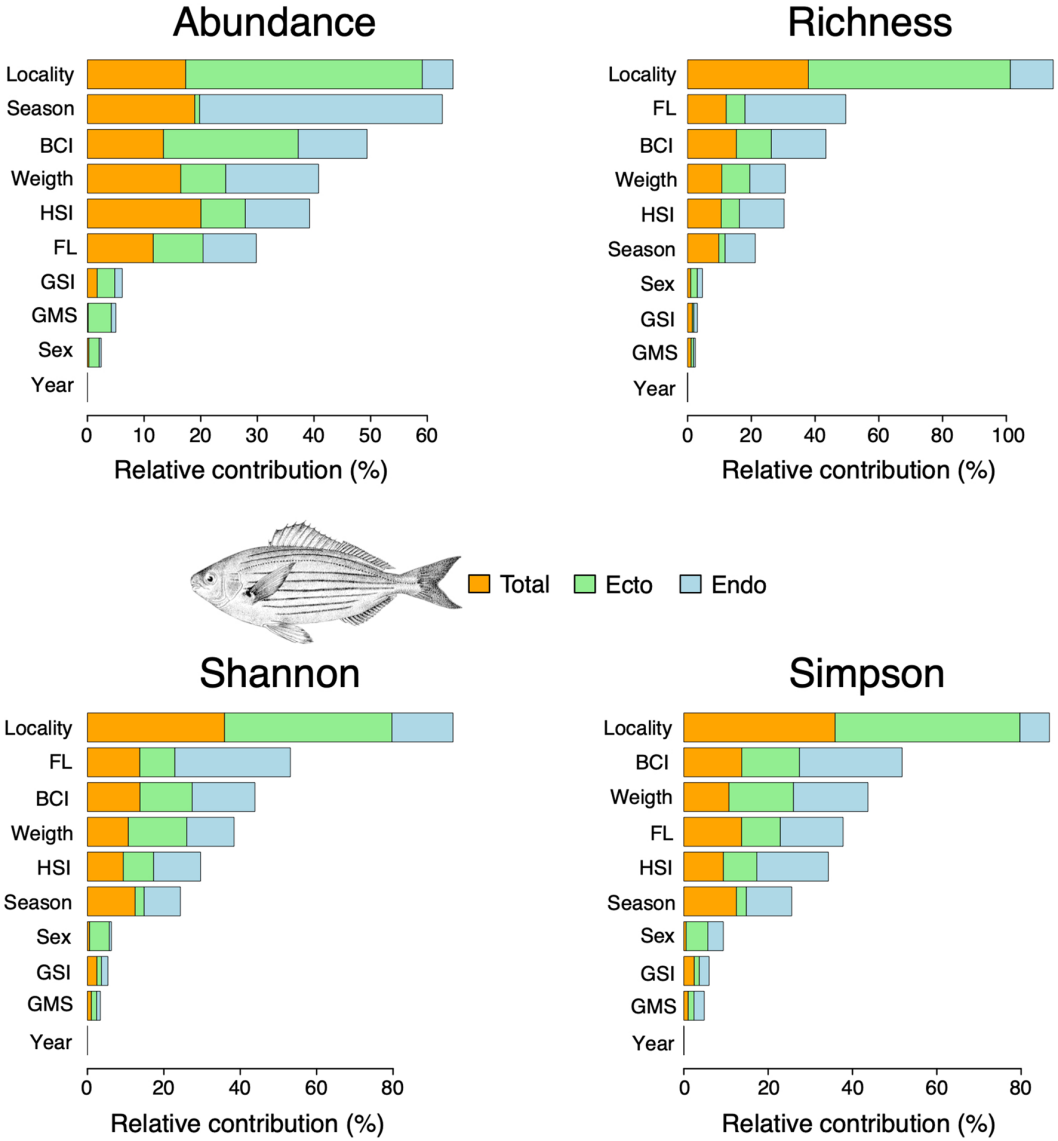
The relative contribution of locality, season, year, and biological (BCI, weight, HSI, FL, GSI, GMS and sex) predictors on parasite abundance, richness, Shannon and Simpson indices of salema *Sarpa salpa* resulting from the boosted regression tree model (BRTm). BCI: body condition index; HSI: hepatosomatic index; FL: fork length; GSI: gonadosomatic index; GMS: gonadal maturity score. The salema drawing in this figure has been obtained from Schneider^51^ and downloaded by https://fishbase.org.

Overall and across all parasite groups, sampling localities were the most important predictors in salema (Table 4, Fig. 2). This was especially true for the ecto-parasite community, which showed higher values in terms of abundance, species richness and Shannon diversity in the GS (Fig. 2 and Supplementary figures S2, S5 and S8). Seasonality was the main predictor of endo-parasite abundance (Fig. 2 and Supplementary figure S3), while size-related factors (e.g. FL, weight and BCI) explained most of the variation in species richness and diversity (Fig. 2 and Supplementary figures S6 and S9). FL showed a step-like relationship with both species’ richness and diversity, characterized by a sudden decrease for intermediate values of FL followed by a *plateau* (Fig. 2 and Supplementary figures S6 and S7), suggesting a given stability in terms of number and diversity of endo-parasites harboured by small-sized individuals. Overall, factors related to sexual maturity (e.g. gonadal stage, HSI and GSI) were less reliable predictors of the parasitic load in salema (Fig. 2).

**Table 4:** Mann–Whitney statistic (*U*) and *p* values (in bold those with significance *p* < 0.05) of the differences between parasite community descriptors from the same host species on different localities (Gulf of Naples *vs*. Gulf of Salerno).

Values	*Sarpa salpa*		*Diplodus sargus*	
	*U*	*p*	*U*	*p*
Total abundance	1,511	** < 0.001**	1,316	0.597
Endo-parasite abundance	1,230	0.075	1,194	0.695
Ecto-parasite abundance	1,768	** < 0.001**	1,404	0.249
Total species richness	1,635	** < 0.001**	1,443	0.105
Endo-parasite species richness	1,285	**0.023**	1,202	0.748
Ecto-parasite species richness	1,754	** < 0.001**	1,535	**0.032**
Total Shannon index	1,577	** < 0.001**	1,380	0.208
Endo-parasite Shannon index	1,299	**0.017**	1,173	0.264
Ecto-parasite Shannon index	1,626	** < 0.001**	1,324	0.466
Total Simpson index	1,111	0.423	1,068	0.227
Endo-parasite Simpson index	929	0.5	1,242	0.986
Ecto-parasite Simpson index	700	** < 0.01**	898	** < 0.001**

**Figure 3:**
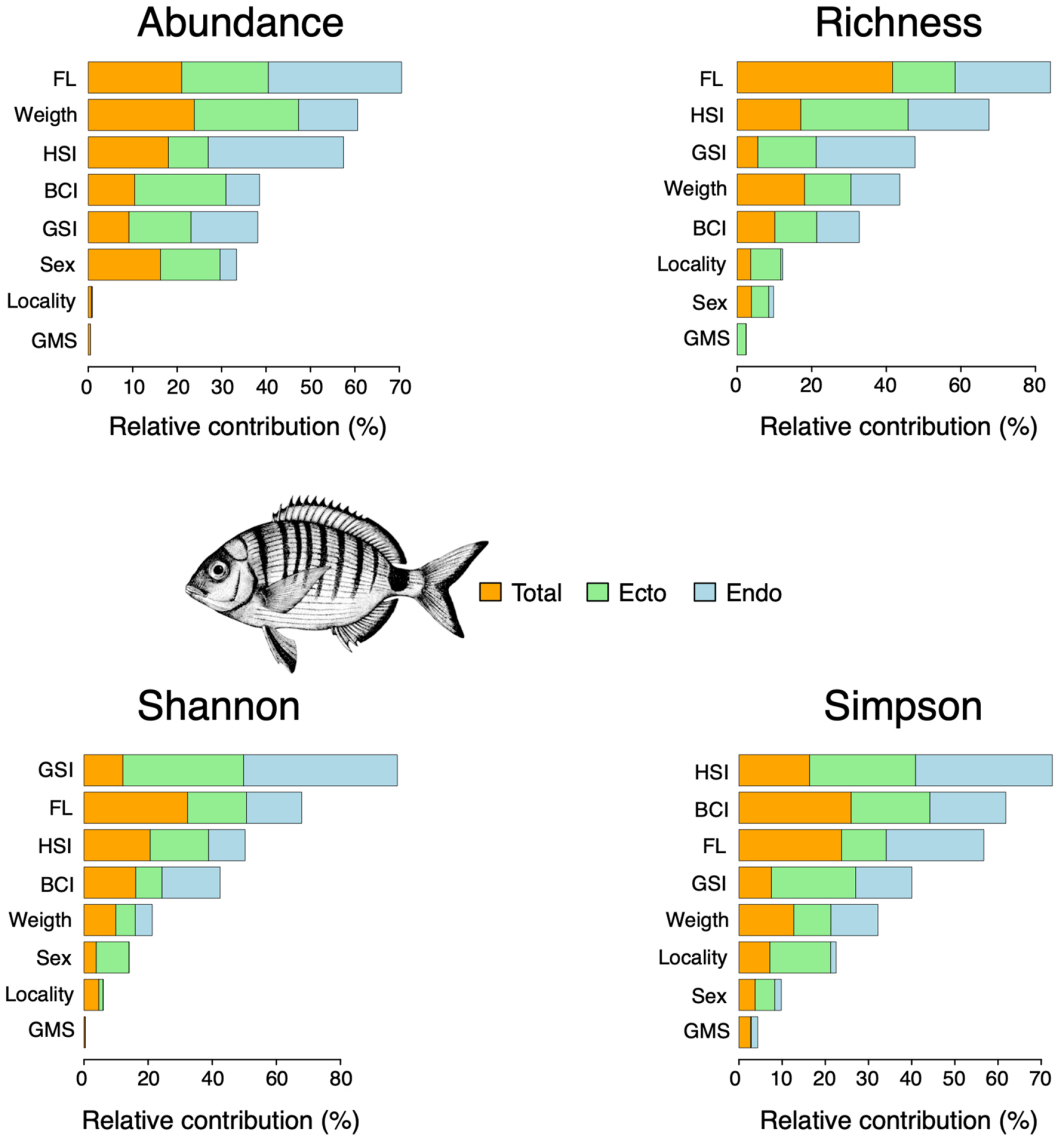
The relative contribution of locality and biological (BCI, weight, HSI, FL, GSI, GMS and sex) predictors on parasite abundance, richness, Shannon and Simpson indices of white seabream *Diplodus sargus* resulting from the boosted regression tree model (BRTm). BCI: body condition index; HSI: hepatosomatic index; FL: fork length; GSI: gonadosomatic index; GMS: gonadal maturity score. The white seabream drawing in this figure has been obtained from Bauchot^52^ and downloaded by https://fishbase.org.

In white seabream, size-related factors (e.g. FL and weight) were the most important predictors for the overall parasite abundance (Fig. 3), with larger individuals harbouring a higher number of parasite individuals. Covariates of size-related factors were largely positively associated with parasite abundance, although some non-linear relationships can be observed in the partial dependence plots (Supplementary figure S13). Besides FL, factors related to the host reproductive cycle were important predictors for parasite richness (Fig. 3). A positive linear relationship was found between FL and GSI of white seabream and endo-parasite species richness, suggesting how large, sexually mature individuals harboured a high number of endo-parasites species (Supplementary figure S18). HSI was the main predictor for ecto-parasite richness (Fig. 3), following a non-linear relationship with a *U*-shaped partial dependence plot characterized by lower values of richness for intermediated HSI (Supplementary figure S17). Ecto-parasite diversity was mainly predicted by factors related to the reproductive cycle, observing a negative linear relationship with GSI and a non-linear U-shaped relationship with HSI (Supplementary figure S20). GSI and, to a lesser extent, FL were the main predictors of endo-parasite diversity in white seabream (Fig. 3), both characterized by a sudden increase followed by a *plateau* (i.e. a step-like distribution). This suggests a threshold limit (independently of sex) in the dimension and sexual maturity of individuals beyond which parasite diversity stabilizes (Supplementary figures S21 and S24).

The most important predictors of parasite community descriptors in salema and white seabream (Figs. 2 and 3) produced relatively strong interaction effects on the abundance of the overall, ecto- and endo-parasite communities. In particular, factor locality in salema had a relatively strong interaction effect with season (H-statistic 0.25), a lower effect with both FL and HSI (H-statistic 0.015 and 0.021, respectively) and only a marginal effect with BCI (H-statistic 0.001). The interaction effect between biological factors was mostly negligible (H-statistic < 0.006), with the only exception for the interaction between HSI and weight (H-statistic 0.1).

Conversely, in white seabream biological factors were the only having strong interaction effects. Weight was found to have a relatively high interaction with both FL and GSI (H-statistic 0.24 and 0.14, respectively) and only marginal with HSI (H-statistic 0.03). Sex had an interaction effect of 0.19 with GSI and only marginal with FL and BCI (H-statistic 0.08 and 0.02, respectively). Other interaction effects between biological factors in white seabream were only negligible (H-statistic < 0.01). The strength of interactions between covariates in BRT models for the Shannon and Simpson indices of both species was near zero when compared with other descriptors.

## Discussion

Parasite communities of salema and white seabream seem to respond differently to host biological factors and sampling locality. The endo-helminths of salema are classified into two families of digeneans restricted to this fish species^27^. In the present study, we found four species of Mesometridae (*Mesometra brachycoelia*, *M. orbicularis*, *Elstia stossichianum*, and *Wardula capitellata*) and one of Gyliauchenidae (*Robphildollfusium fractum*). First intermediate hosts of Mesometridae are gastropod molluscs (Prosobranchia); infection of the definitive host occurs by ingestion of cercariae encysted on algae or marine flowering plants^28^. No data exists on the life cycle of members within Gyliauchenidae, however the concentration of its members in herbivorous fishes strongly suggests that the life cycle might incorporate a cercaria that encysts on algae, as it has been shown for Mesometridae^28,29^.

The endo-helminths of white seabream are largely generalist parasites. In the present study we found only two common parasite species in both localities (prevalence ≤ 16%): the nematode *Cucullanus campanae* (Cucullanidae) and the digenean *Lepocreadium pegorchys* (Lepocreadiidae). Infective larvae of *Cucullanus* spp. develop in polychaetes and copepods^30^, while *Lepocreadium* spp. use sorbeoconchan gastropods as first intermediate host, and a wide range of invertebrates including medusa, ctenophores, polychaetes, turbellarians as second intermediate hosts^31^. Ecto-parasites of both fish hosts included members within three main groups (leeches, crustaceans and monogeneans) with different ecological and biological features, however all characterized by direct life cycle.

When comparing descriptors of communities between the two fish hosts, we found significant differences only in endo-parasites, with the herbivorous-feeding salema showing higher values (Table 3). Results from studies of helminth communities of fishes show that the richest enteric helminth assemblages are found in carnivorous fishes, whereas algal feeders, herbivores and detritivores showed species poor helminth communities^32,33^. The results presented in this study are in apparent deviation from our expectations, considering that helminth communities of white seabream in other Mediterranean areas are richest than that found here^33–35^. To explain this finding, we hypothesize that selective feeding on specific poorly infected food items in sampling areas may have led to the poor endo-parasites communities in the white seabream. Similarly, a study from the Tyrrhenian coast of southern Italy showed that in the last decade, the white seabream changed its feeding habit having become the invasive green alga *Caulerpa racemosa* the most important component of its diet^36^. In addition, Bartoli et al.^37^ suggested for the labrid fish *Syrnphodus ocellatus* living in sites colonized by the invasive alga *Caulerpa taxifolia* that secondary metabolites as caulerpenyne and other terpenes synthesized by this alga and released into the environment or transmitted along the food web, might be responsible for the near-complete disappearance of digeneans in *S. ocellatus*. In the present study, we did not focus the attention on the quantification and identification to the lowest taxonomic level of the gastro-intestinal contents. However, gross analysis of gastro-intestinal material during the parasitological analysis revealed that remains of *Caulerpa* spp. were common food items found in the white seabream from both localities, strongly supporting the hypothesis regarding the possible depletion of digenean community due to the new acquired feeding habit.

Geographical features and, possibly, the degree of alteration of the two studied basins could account for differences in the parasite communities found in salema and, partially, white seabream (only for the ecto-parasites), with the GN showing poorest communities. Parasite communities may be good indicators of environmental disturbance because they reflect the interactions between a possible stressor and either free-living larval stages or populations of their intermediate and final hosts^2–4,9,10,13^. The Gulf of Castellammare in the south-eastern part of GN (where fish samples were obtained) is strongly affected by the sewages of the Sarno River^14–16^. The poor values of communities in GN suggest an unstable ecosystem with a decrease in the biomass and densities of hosts. In contrast, the higher values of parasite communities found in GS, where samples were obtained close to a marine reserve, could be related to the relative stability of its ecosystem^2,3,11–13^, which shows abundant *Posidonia* meadows and a more preserved benthonic ecosystem with richer communities of intermediate hosts for digeneans. Regarding the ecto-parasite community, it has been demonstrated that crustaceans and monogeneans are more sensitive than endo-parasites to the environmental deterioration, anthropogenic or other pressures (e.g. high levels of conductivity, nutrients and hypoxia)^2,4,6,7^. This is because they are directly exposed to water and to environmental changes and may be strongly affected by reducing their survival and reproduction rates ^2,4,6,7^.

For instance, Kostarev^38^ observed that industrial waste discharge into two reservoirs affected negatively the species richness of monogeneans in fishes. Similarly, it has been documented that the prevalence and abundance of crustacean parasites decreased considerably in polluted sites^39,40^, and no ecto-parasites have been found on fishes at the closest location to the effluent discharge^40^ or at polluted sites when compared with un-polluted sites^41,42^.

In general, size-related factors including BCI, FL and weight explained most of predictor variables in parasite communities for both host species, and interaction effects suggested the influence of localities and season on specific biological traits in salema but not in white seabream, ultimately influencing the abundance of both ecto- and endo-parasites. FL was the most important predictor for species richness and diversity of endo-parasite community in salema and for the overall parasite abundance and species richness in the white seabream*,* respectively. Our results are in accordance with previous investigations^8,43–46^, which suggested that larger body size increases the vagility and habitat exploitation by hosts^8,47,48^, therefore making them a better target for the infective stage of a parasite and enhancing the exposure to more parasite species on a regional scale. In white seabream, the ecto-parasite diversity was also predicted by factors related to the reproductive cycle, supporting the view that the vagility and aggregation of individuals during the reproductive season may facilitate the infection of parasites with direct life-cycles, leading to more diverse ecto-parasite assemblages^5,47–50^. Moreover, in salema, seasonality was the main predictor of endo-parasite abundance with highest value in winter (December/February), suggesting the seasonal variation in availability of encysted larval digeneans.

## Conclusion

The present study shows that the structure of parasite community of salema and white seabream from two contiguous areas is the result of complex interactions between environment, fish host populations and free-living larval stages or populations of their intermediate hosts. Host size-related factors are important natural predictors of parasite community in both host species. However, geographic locality may affect differently the parasite community depending on host species and sensitivity of their parasite stages to environmental features and their changes and pressures. Compared to GS, the poorer parasite communities found in GN suggests that changes and deterioration of environmental conditions can play an important role, affecting the parasite communities of these fishes, that in turn, could be used as biological indicators. However, further investigations are needed to provide conclusive data about the nature and importance of the potential effects of environmental degradation, by studying the temporal fluctuation of parasite community descriptors in these common sparid fishes.

## Supplementary information

Supplementary figures S1-S12

Supplementary figures S13-S24
