# Supplementary material for: Host biological factors and geographic locality influence predictors of parasite communities in sympatric sparid fishes off the southern Italian coast
Source: Sci Rep. 2020 Aug 6;10:13283. doi: 10.1038/s41598-020-69628-1 (PMC7414025; doi:10.1038/s41598-020-69628-1)
Supplement: Supplementary file 1 — Supplementary figures S1-S12 [file 41598_2020_69628_MOESM1_ESM.pdf]

Supplementary materials

Host biological factors and geographic locality influence predictors of parasite communities in sympatric sparid fishes off the southern Italian coast

Mario Santoro[1,*], Doriana Iaccarino[2], Bruno Bellisario[3,4,5]

[1]Stazione Zoologica Anton Dohrn, Department of Integrative Marine Ecology, Villa Comunale 1, Naples, 80121, Italy

[2]Istituto Zooprofilattico Sperimentale del Mezzogiorno, Department of Animal Health, Via Salute 9, Portici, 80055, Italy

[3]University of Minho, Centre of Molecular and Environmental Biology, Campus de Gualtar, Braga, 4710-057, Portugal

[4]University of Minho, Institute of Science and Innovation for Bio-Sustainability, Campus de Gualtar, Braga, 4710-057, Portugal

[5]Department of Ecological and Biological Sciences, Largo dell'Università Snc, University of Viterbo, 01100, Italy

*Correspondence to mario.santoro@szn.it

Variables importance in boosted regression tree models

In this supplementary material, we show for each group (total, ecto- and endo-parasite) the partial dependence plots for all non-zero predictive variables in models of abundance, richness, Shannon and Simpson diversity for the salema *Sarpa salpa* (Figs S1-S12).

Figure S1 -

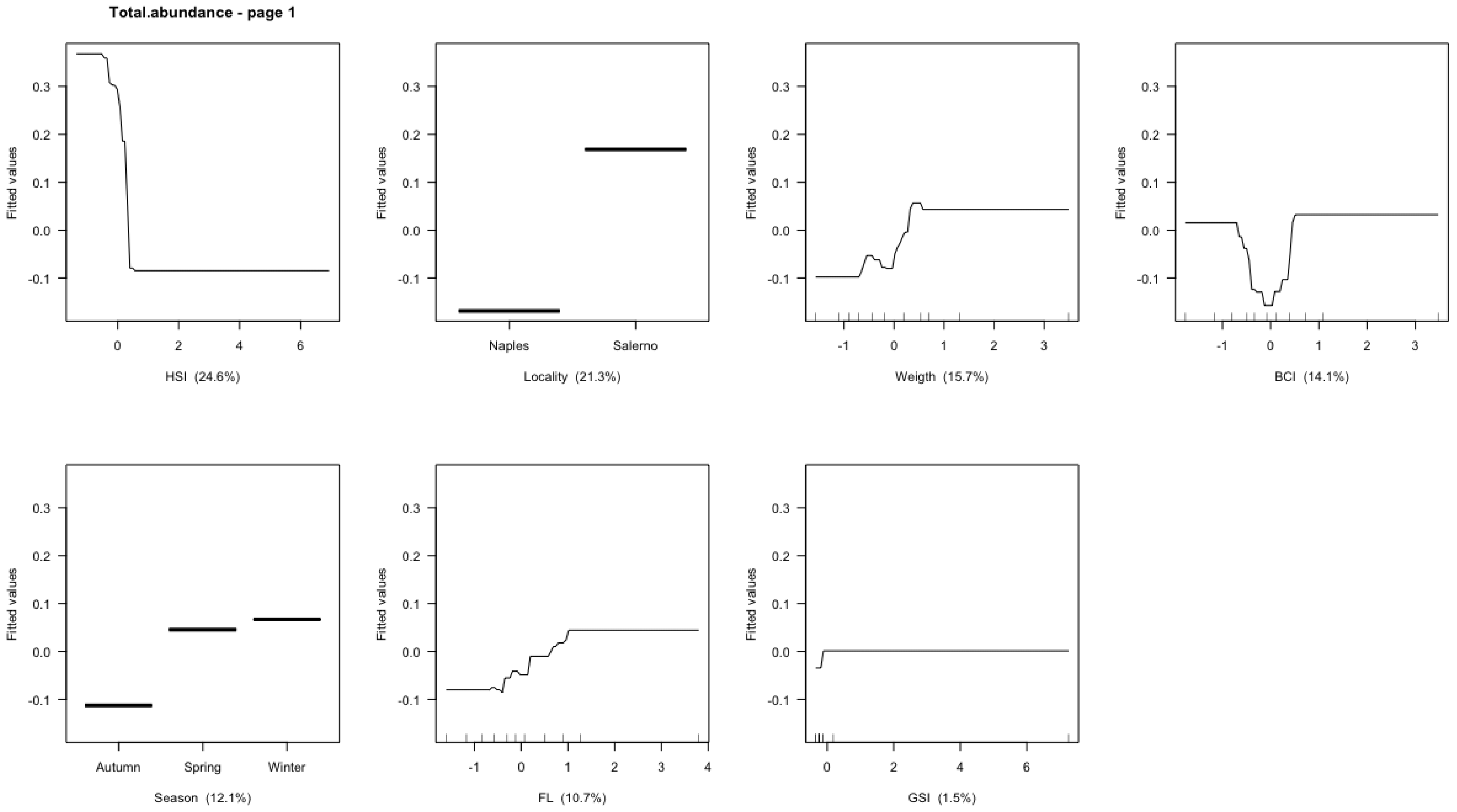
Total.abundance - page 1
Fitted values
HSI (24.6%)
Locality (21.3%)
Naples
Salerno
Weigth (15.7%)
BCI (14.1%)
Season (12.1%)
Autumn
Spring
Winter
FL (10.7%)
GSI (1.5%)

Figure S2 -

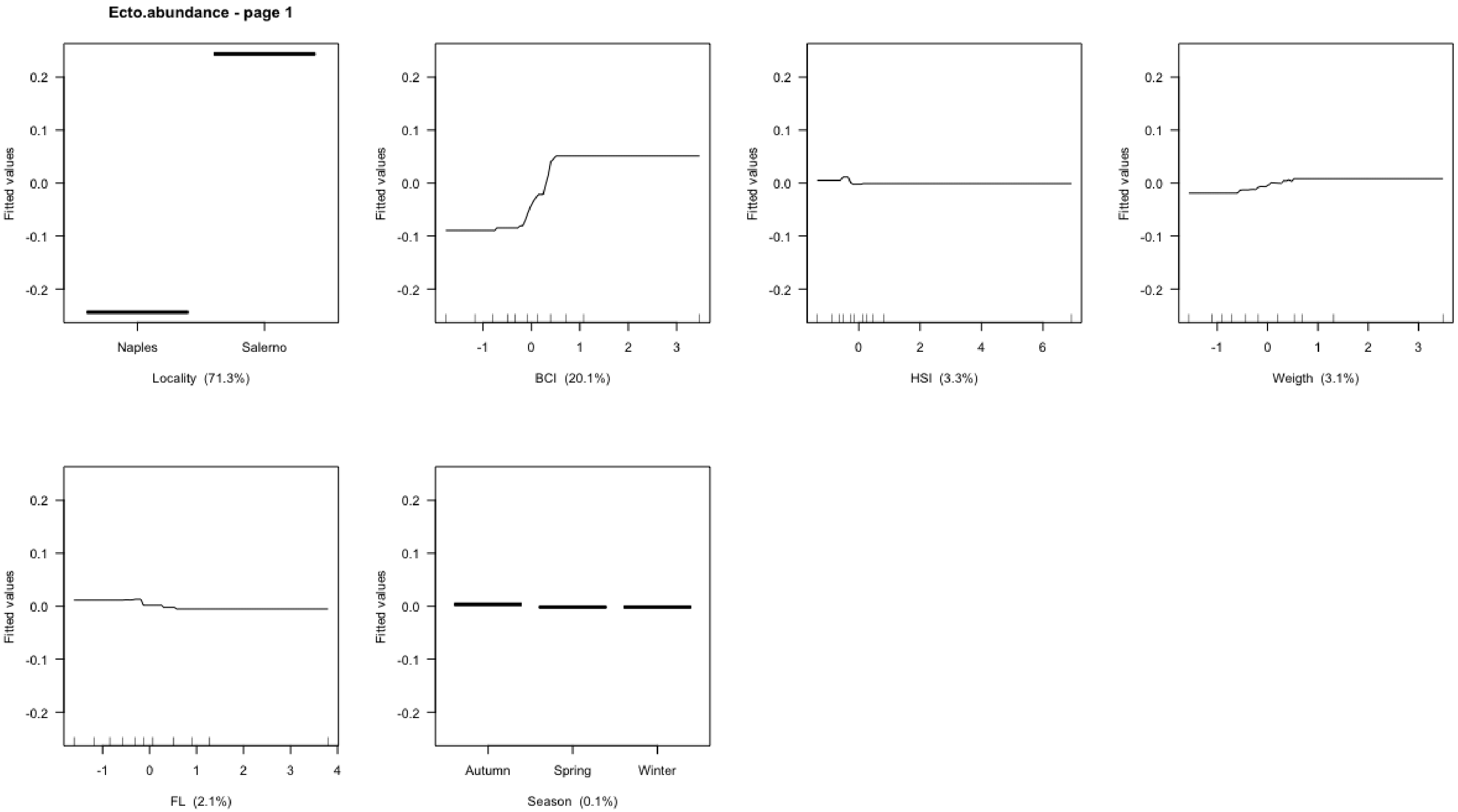

Ecto.abundance - page 1
Fitted values
0.2
0.1
0.0
-0.1
-0.2
Naples
Salerno
Locality (71.3%)
-1
0
1
2
3
BCI (20.1%)
0
2
4
6
HSI (3.3%)
Weigth (3.1%)
4
FL (2.1%)
Autumn
Spring
Winter
Season (0.1%)

Figure S3 -

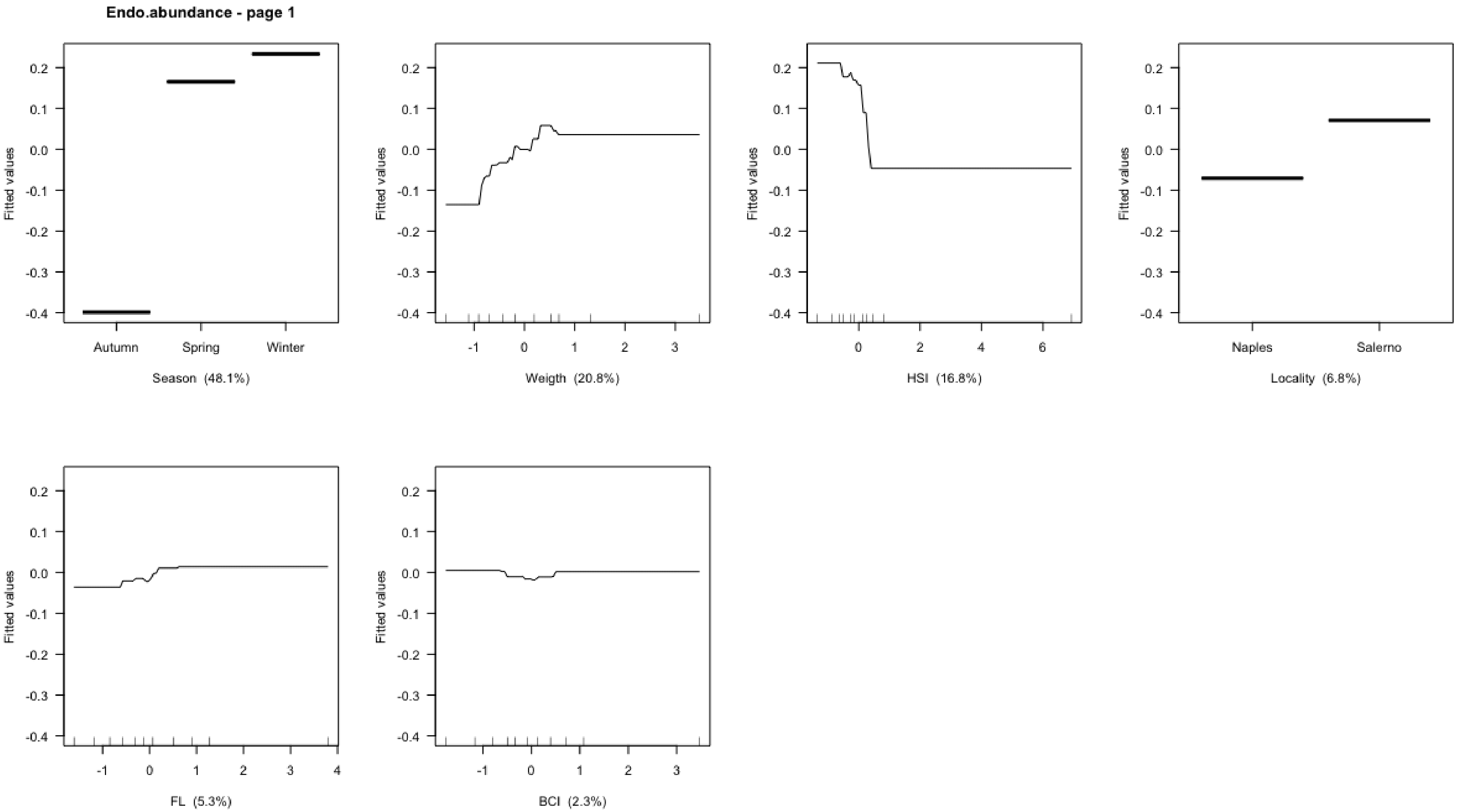
Endo.abundance - page 1
Fitted values
Autumn
Spring
Winter
Season (48.1%)
Weigth (20.8%)
HSI (16.8%)
Naples
Salerno
Locality (6.8%)
FL (5.3%)
BCI (2.3%)

Figure S4 -

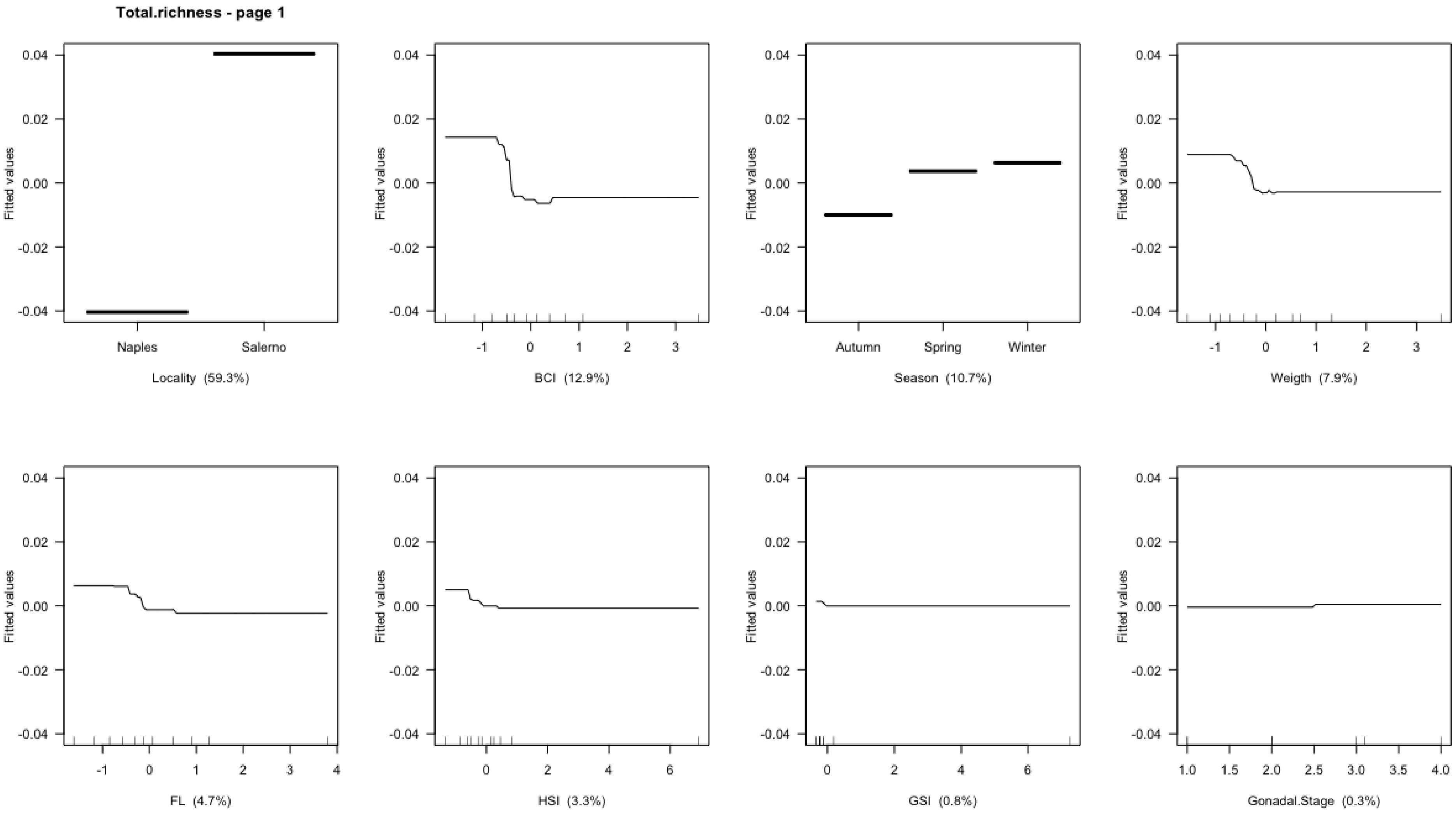

Total.richness - page 1
Fitted values
0.04
0.02
0.00
-0.02
-0.04
Naples
Salerno
Locality (59.3%)
BCI (12.9%)
Autumn
Spring
Winter
Season (10.7%)
Weigth (7.9%)
FL (4.7%)
HSI (3.3%)
GSI (0.8%)
Gonadal.Stage (0.3%)

Figure S5 -

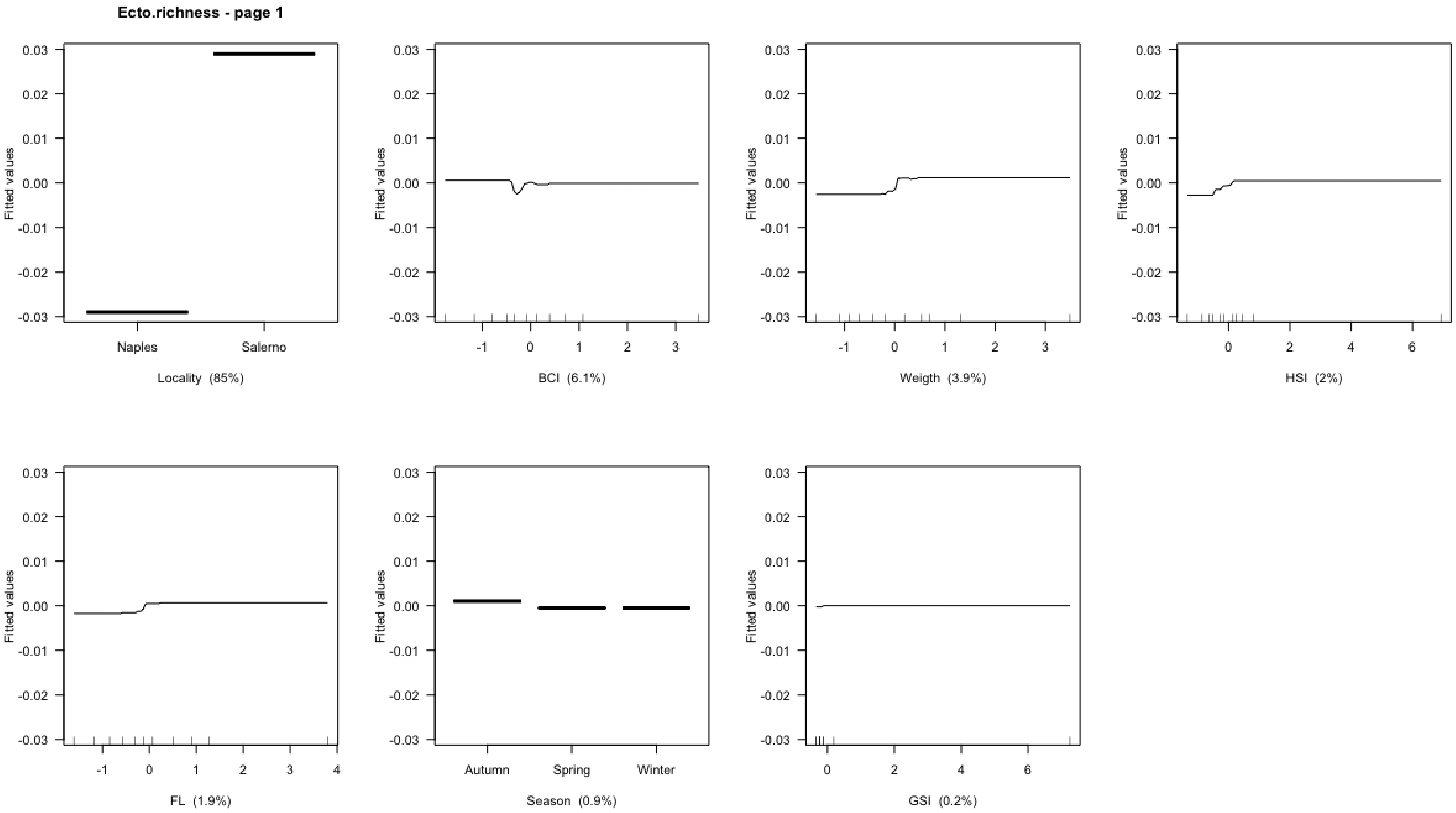
Ecto.richness - page 1
Fitted values
Locality (85%)
Naples
Salerno
BCI (6.1%)
Weigth (3.9%)
HSI (2%)
FL (1.9%)
Season (0.9%)
Autumn
Spring
Winter
GSI (0.2%)

Figure S6 -

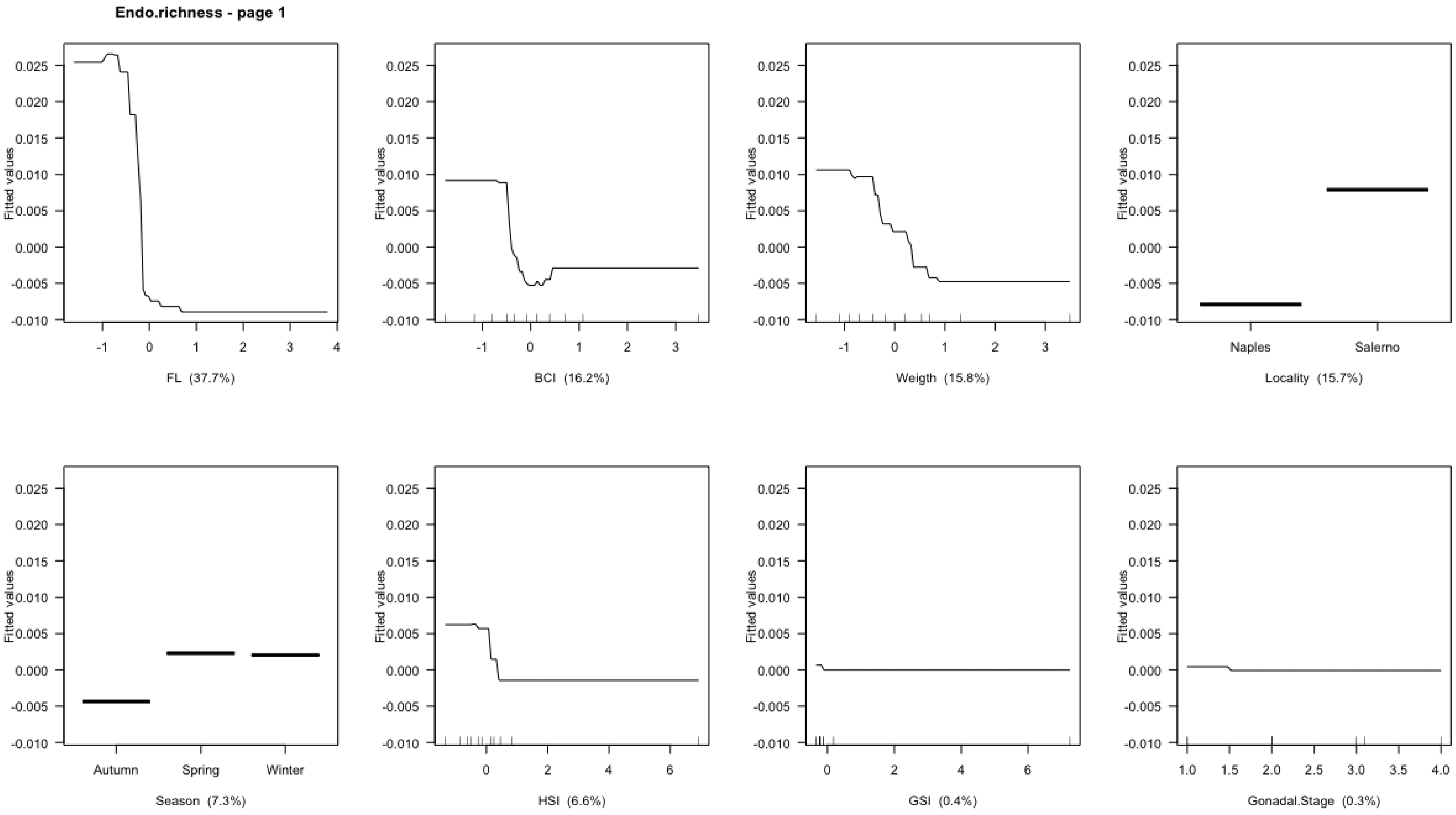

Endo.richness - page 1
Fitted values
FL (37.7%)
BCI (16.2%)
Weigth (15.8%)
Locality (15.7%)
Naples
Salerno
Season (7.3%)
Autumn
Spring
Winter
HSI (6.6%)
GSI (0.4%)
Gonadal.Stage (0.3%)

Figure S7 -

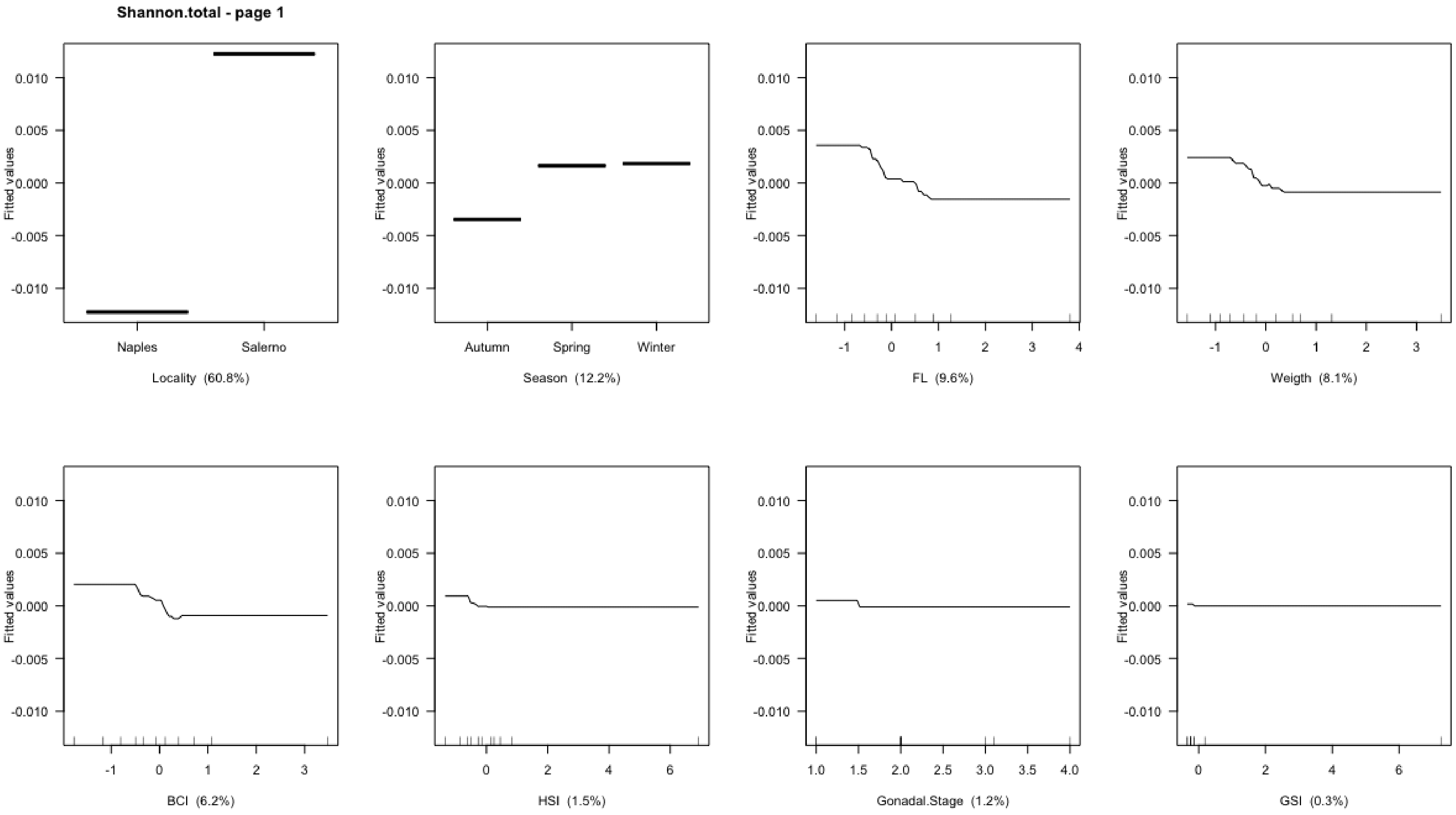
Shannon.total - page 1
Fitted values
Naples
Salerno
Locality (60.8%)
Autumn
Spring
Winter
Season (12.2%)
FL (9.6%)
Weigth (8.1%)
BCI (6.2%)
HSI (1.5%)
Gonadal.Stage (1.2%)
GSI (0.3%)

Figure S8 -

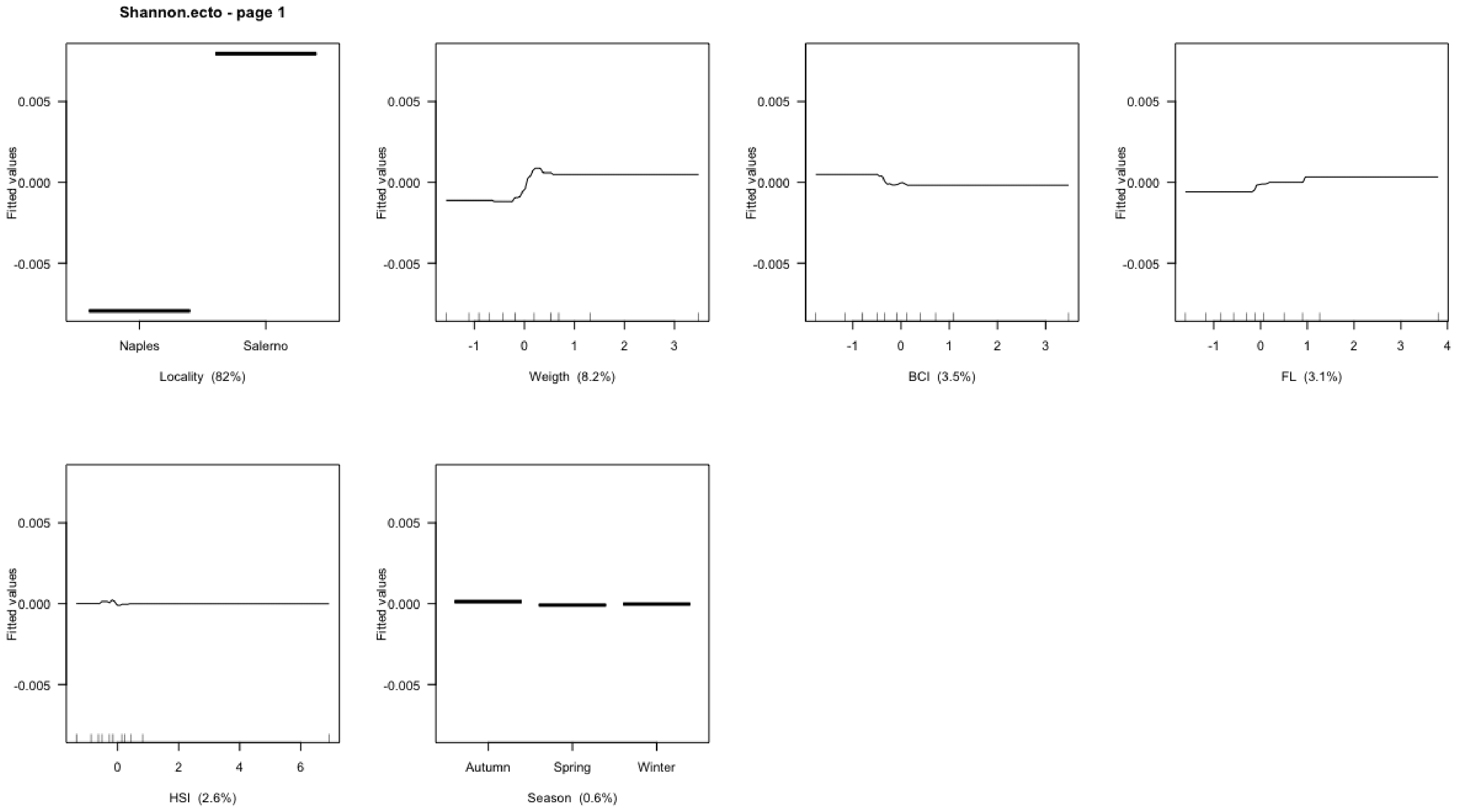
Shannon.ecto - page 1
Fitted values
0.005
0.000
-0.005
Naples
Salerno
Locality (82%)
Weigth (8.2%)
BCI (3.5%)
FL (3.1%)
HSI (2.6%)
Autumn
Spring
Winter
Season (0.6%)

Figure S9 -

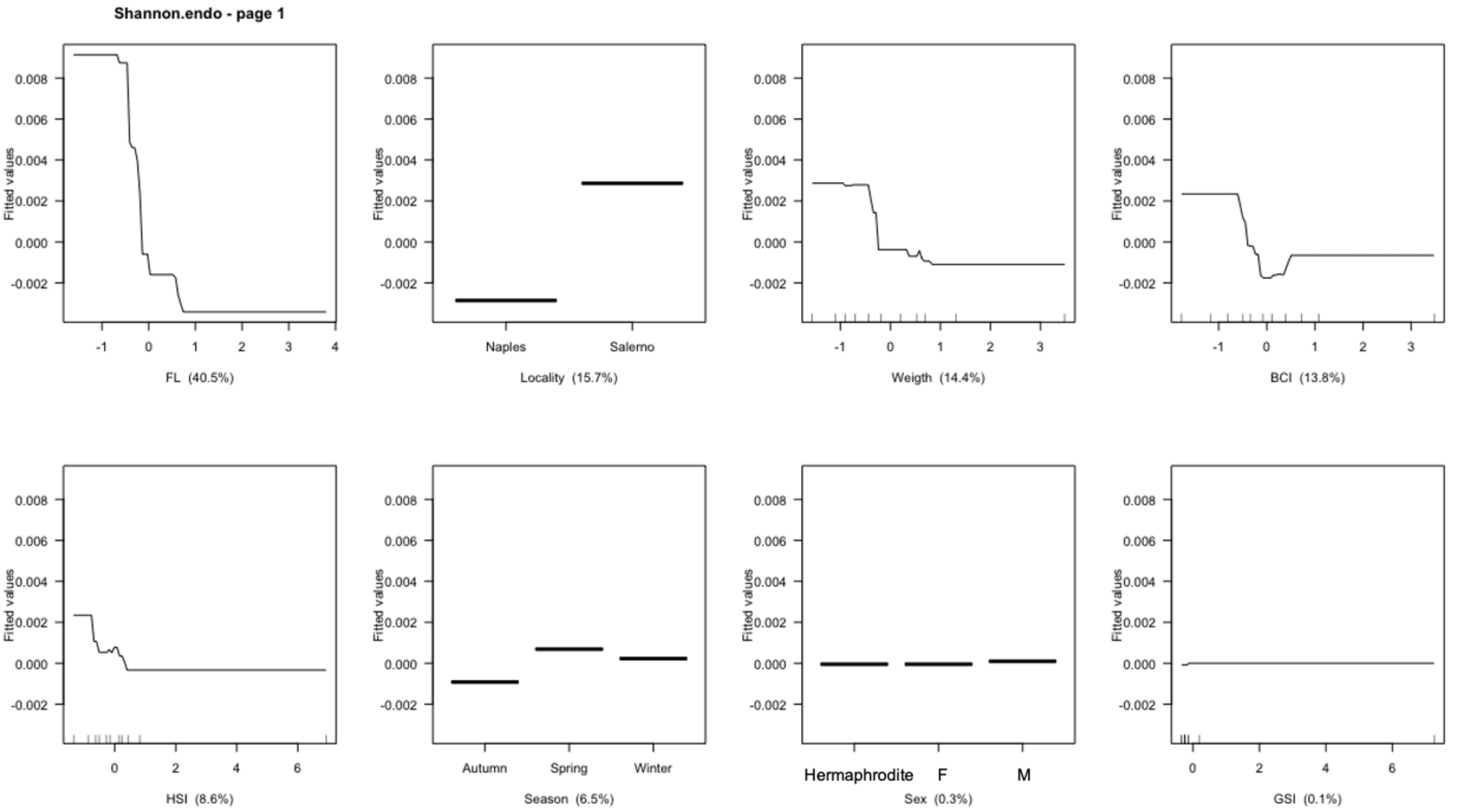
Shannon.endo - page 1
Fitted values
FL (40.5%)
Locality (15.7%)
Naples
Salerno
Weigth (14.4%)
BCI (13.8%)
HSI (8.6%)
Season (6.5%)
Autumn
Spring
Winter
Sex (0.3%)
Hermaphrodite
F
M
GSI (0.1%)

Figure S10 -

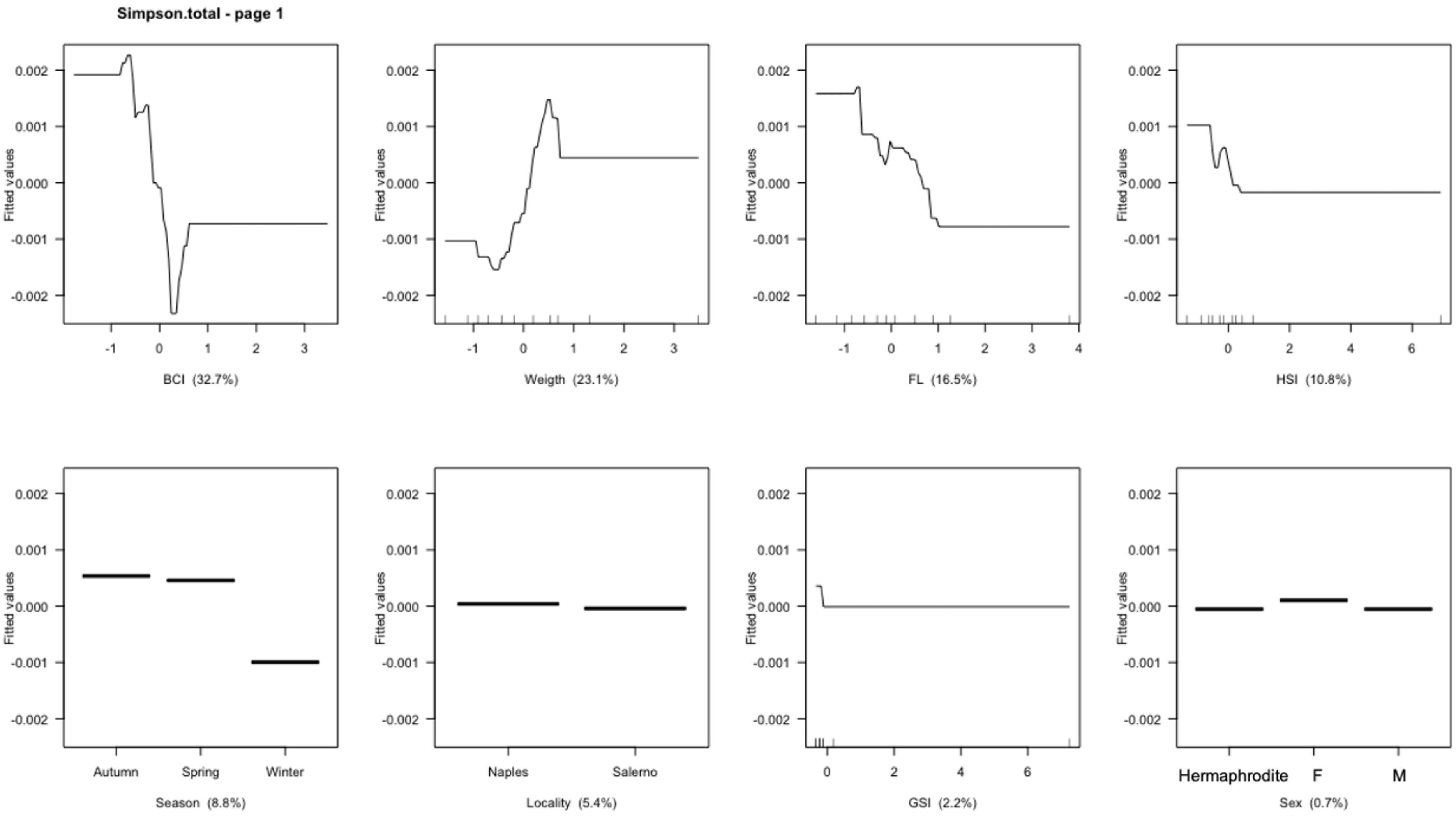

Simpson.total - page 1
Fitted values
0.002
0.001
0.000
-0.001
-0.002
BCI (32.7%)
Weigth (23.1%)
FL (16.5%)
HSI (10.8%)
Autumn
Spring
Winter
Season (8.8%)
Naples
Salerno
Locality (5.4%)
GSI (2.2%)
Hermaphrodite
F
M
Sex (0.7%)

Figure S11 -

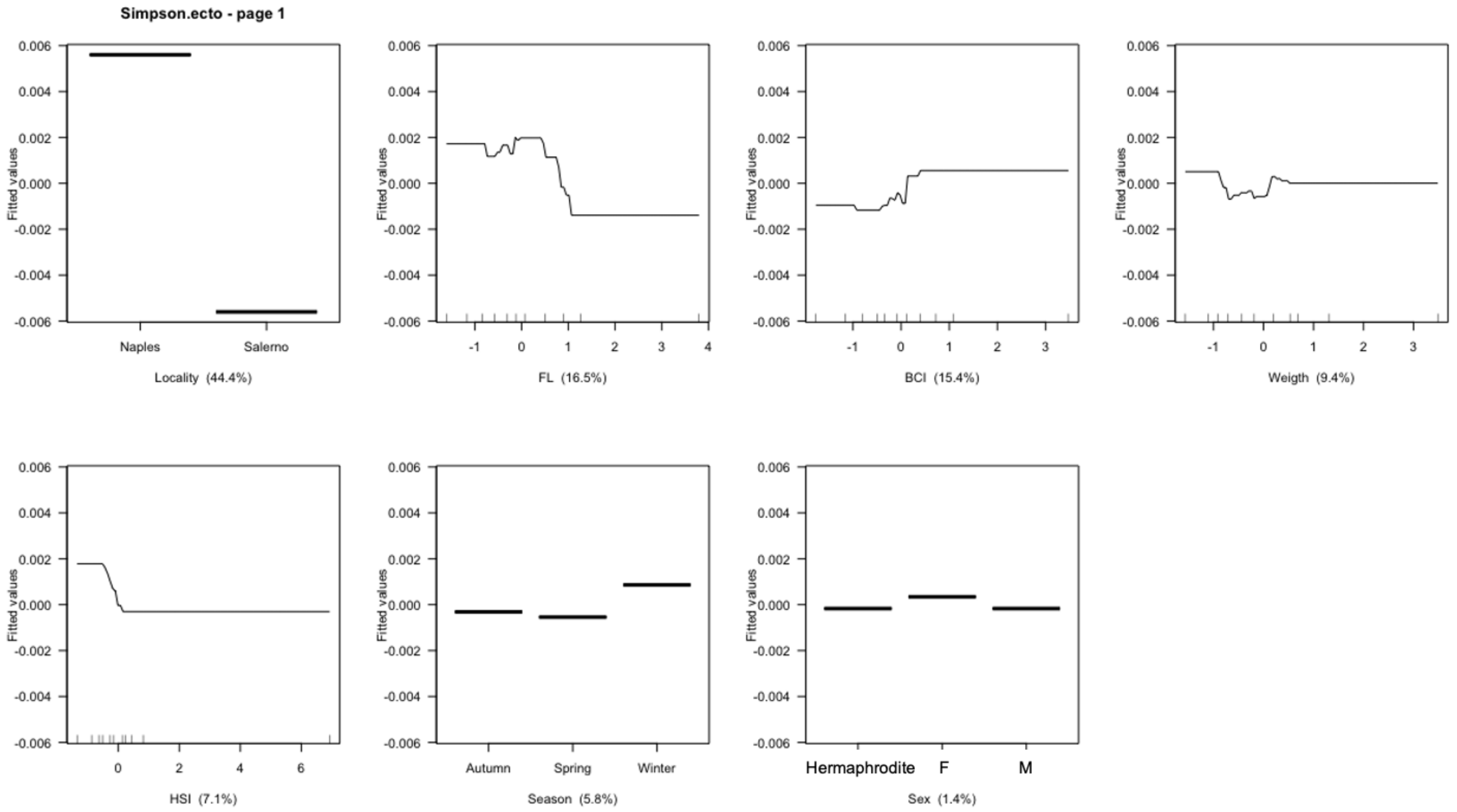
Simpson.ecto - page 1
Fitted values
Naples
Salerno
Locality (44.4%)
FL (16.5%)
BCI (15.4%)
Weigth (9.4%)
HSI (7.1%)
Autumn
Spring
Winter
Season (5.8%)
Hermaphrodite
F
M
Sex (1.4%)

Figure S12 -

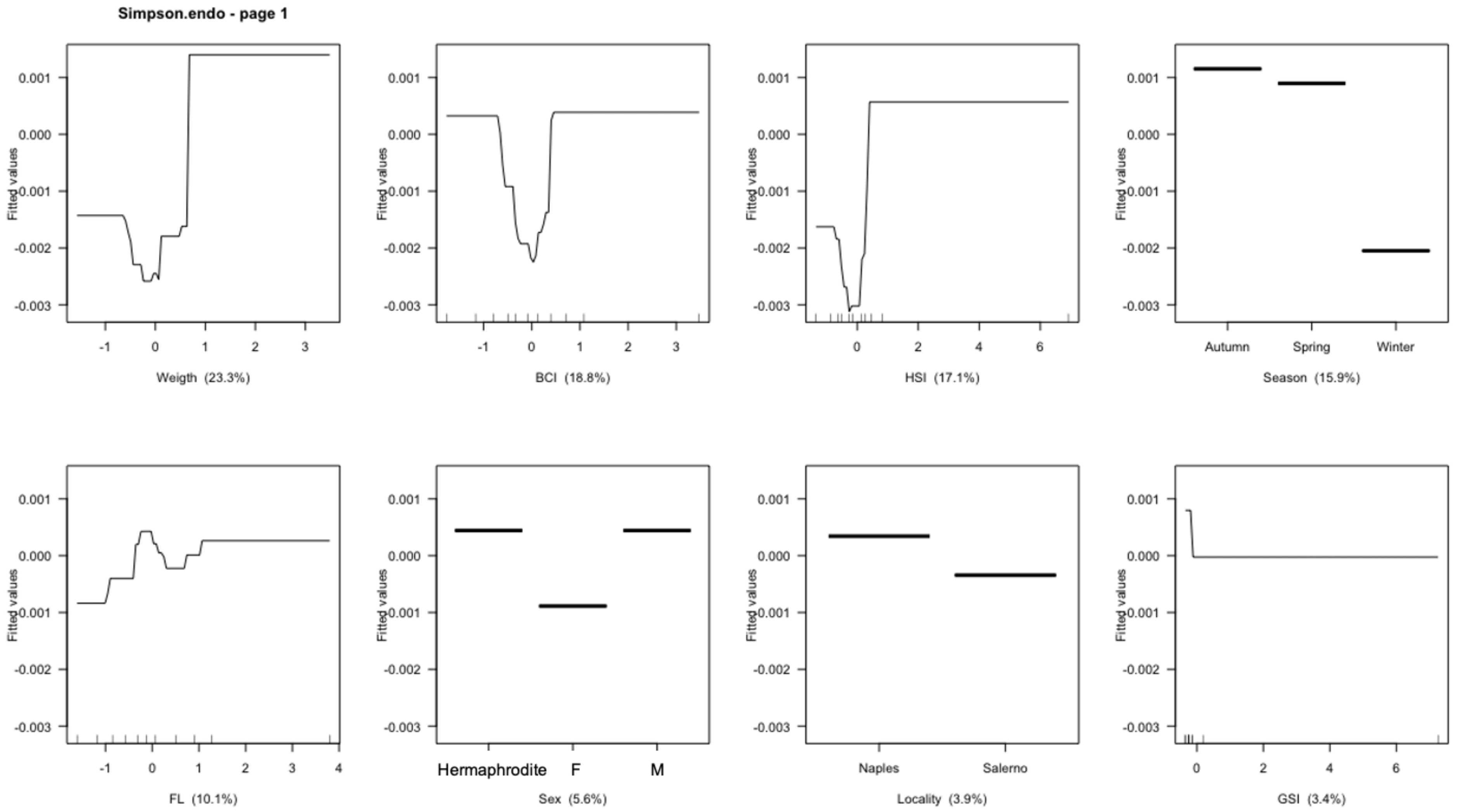
Simpson.endo - page 1
Fitted values
0.001
0.000
-0.001
-0.002
-0.003
Weigth (23.3%)
BCI (18.8%)
HSI (17.1%)
Season (15.9%)
Autumn
Spring
Winter
FL (10.1%)
Sex (5.6%)
Hermaphrodite
F
M
Locality (3.9%)
Naples
Salerno
GSI (3.4%)
