# Supplementary material for: Host biological factors and geographic locality influence predictors of parasite communities in sympatric sparid fishes off the southern Italian coast
Source: Sci Rep. 2020 Aug 6;10:13283. doi: 10.1038/s41598-020-69628-1 (PMC7414025; doi:10.1038/s41598-020-69628-1)
Supplement: Supplementary file 2 — Supplementary figures S13-S24 [file 41598_2020_69628_MOESM2_ESM.pdf]

Supplementary materials

Host biological factors and geographic locality influence predictors of parasite communities in sympatric sparid fishes off the southern Italian coast

Mario Santoro[1,*], Doriana Iaccarino[2], Bruno Bellisario[3,4,5]

[1]Stazione Zoologica Anton Dohrn, Department of Integrative Marine Ecology, Villa Comunale 1, Naples, 80121, Italy

[2]Istituto Zooprofilattico Sperimentale del Mezzogiorno, Department of Animal Health, Via Salute 9, Portici, 80055, Italy

[3]University of Minho, Centre of Molecular and Environmental Biology, Campus de Gualtar, Braga, 4710-057, Portugal

[4]University of Minho, Institute of Science and Innovation for Bio-Sustainability, Campus de Gualtar, Braga, 4710-057, Portugal

[5]Department of Ecological and Biological Sciences, Largo dell'Università Snc, University of Viterbo, 01100, Italy

*Correspondence to mario.santoro@szn.it

Variables importance in boosted regression tree models

In this supplementary material, we show for each group (total, ecto- and endo-parasite) the partial dependence plots for all non-zero predictive variables in models of abundance, richness, Shannon and Simpson diversity for the white seabream *Diplodus sargus* (Figs S13-S24).

Figure S13 -

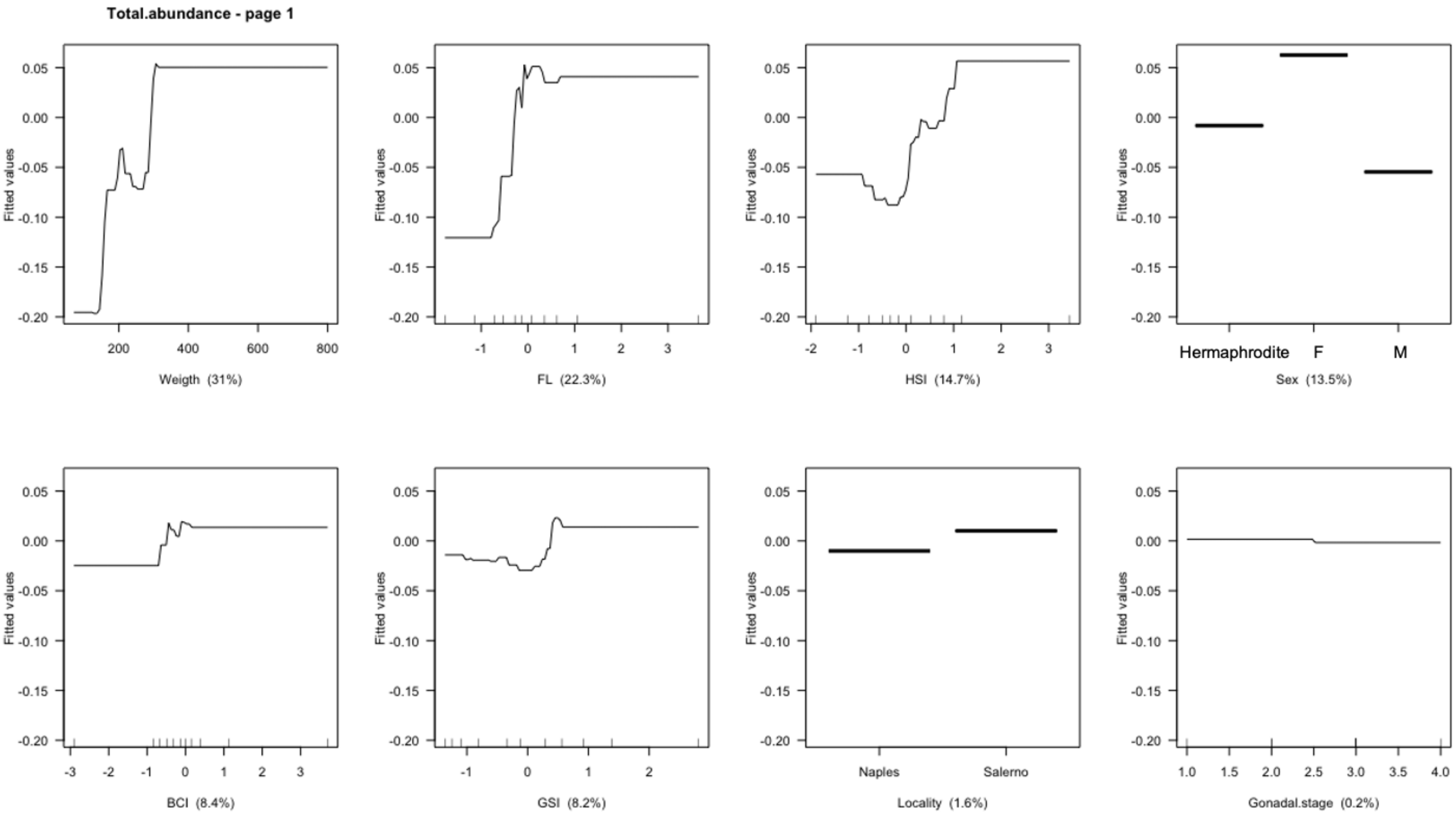

Total.abundance - page 1
Fitted values
Weigth (31%)
FL (22.3%)
HSI (14.7%)
Sex (13.5%)
Hermaphrodite
F
M
BCI (8.4%)
GSI (8.2%)
Locality (1.6%)
Naples
Salerno
Gonadal.stage (0.2%)

Figure S14 -

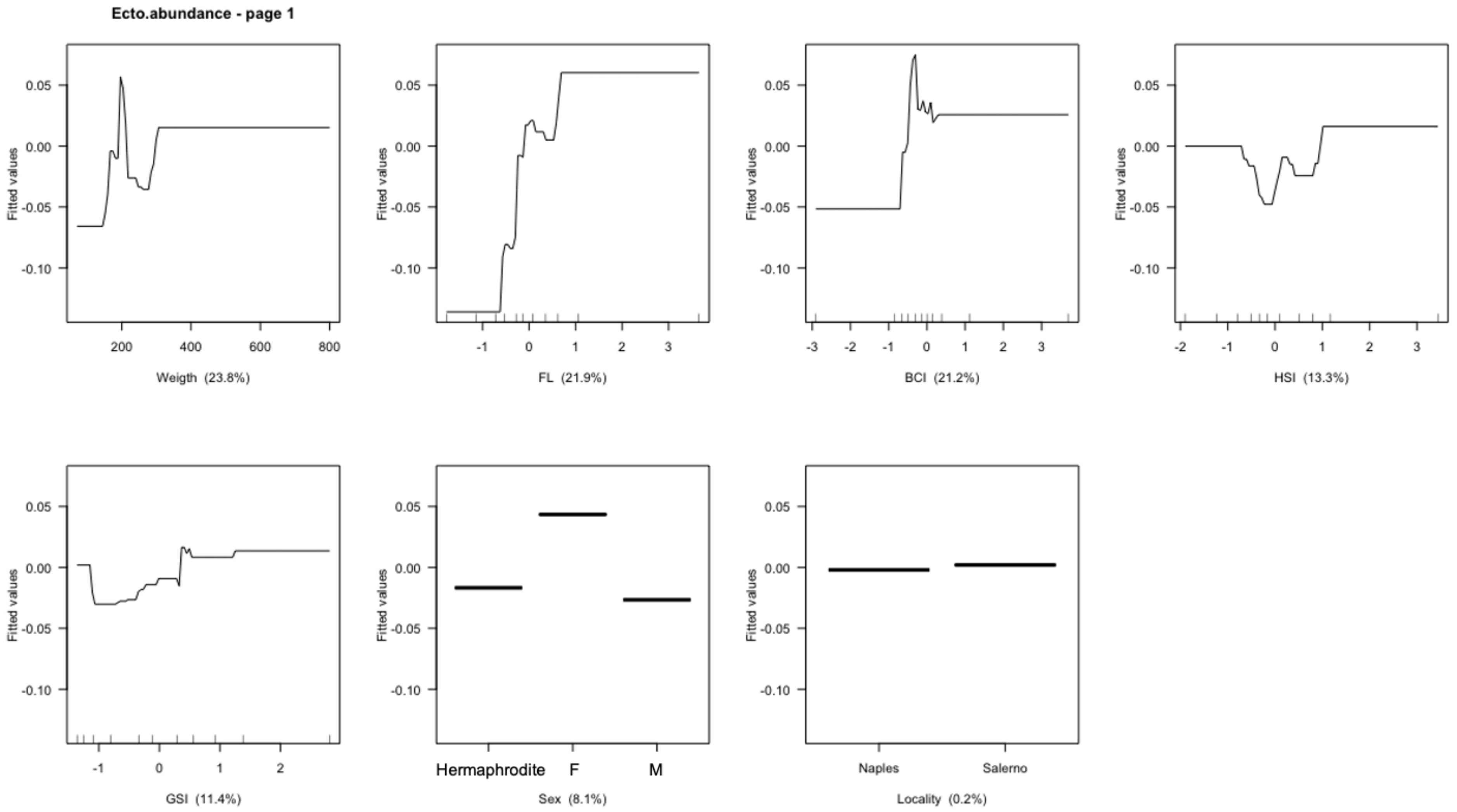
Ecto.abundance - page 1
Fitted values
0.05
0.00
-0.05
-0.10
200
400
600
800
Weigth (23.8%)
-1
0
1
2
3
FL (21.9%)
-3
-2
BCI (21.2%)
HSI (13.3%)
GSI (11.4%)
Hermaphrodite
F
M
Sex (8.1%)
Naples
Salerno
Locality (0.2%)

Figure S15 -

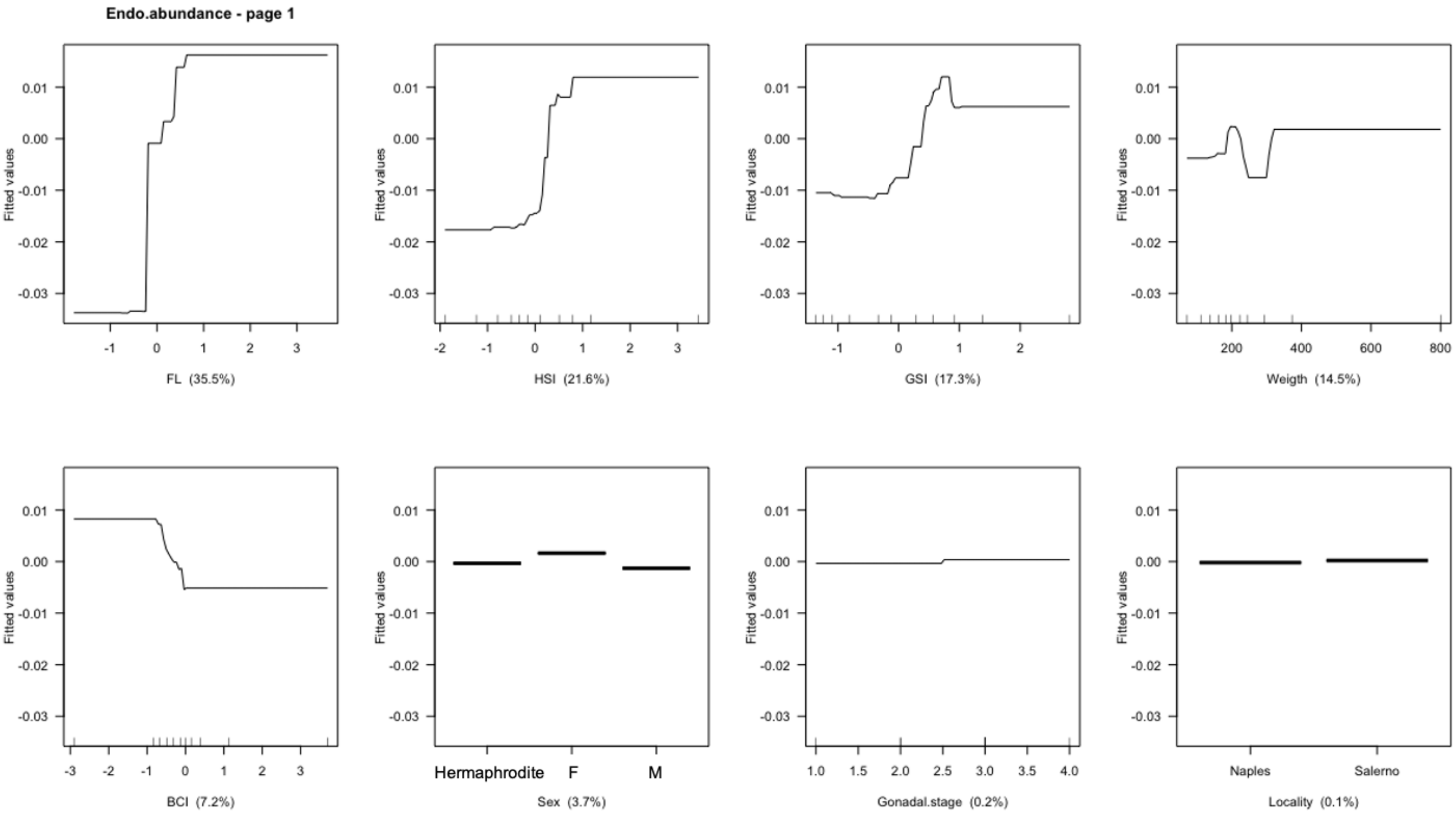
Endo.abundance - page 1
Fitted values
FL (35.5%)
HSI (21.6%)
GSI (17.3%)
Weigth (14.5%)
BCI (7.2%)
Sex (3.7%)
Hermaphrodite
F
M
Gonadal.stage (0.2%)
Locality (0.1%)
Naples
Salerno

Figure S16 -

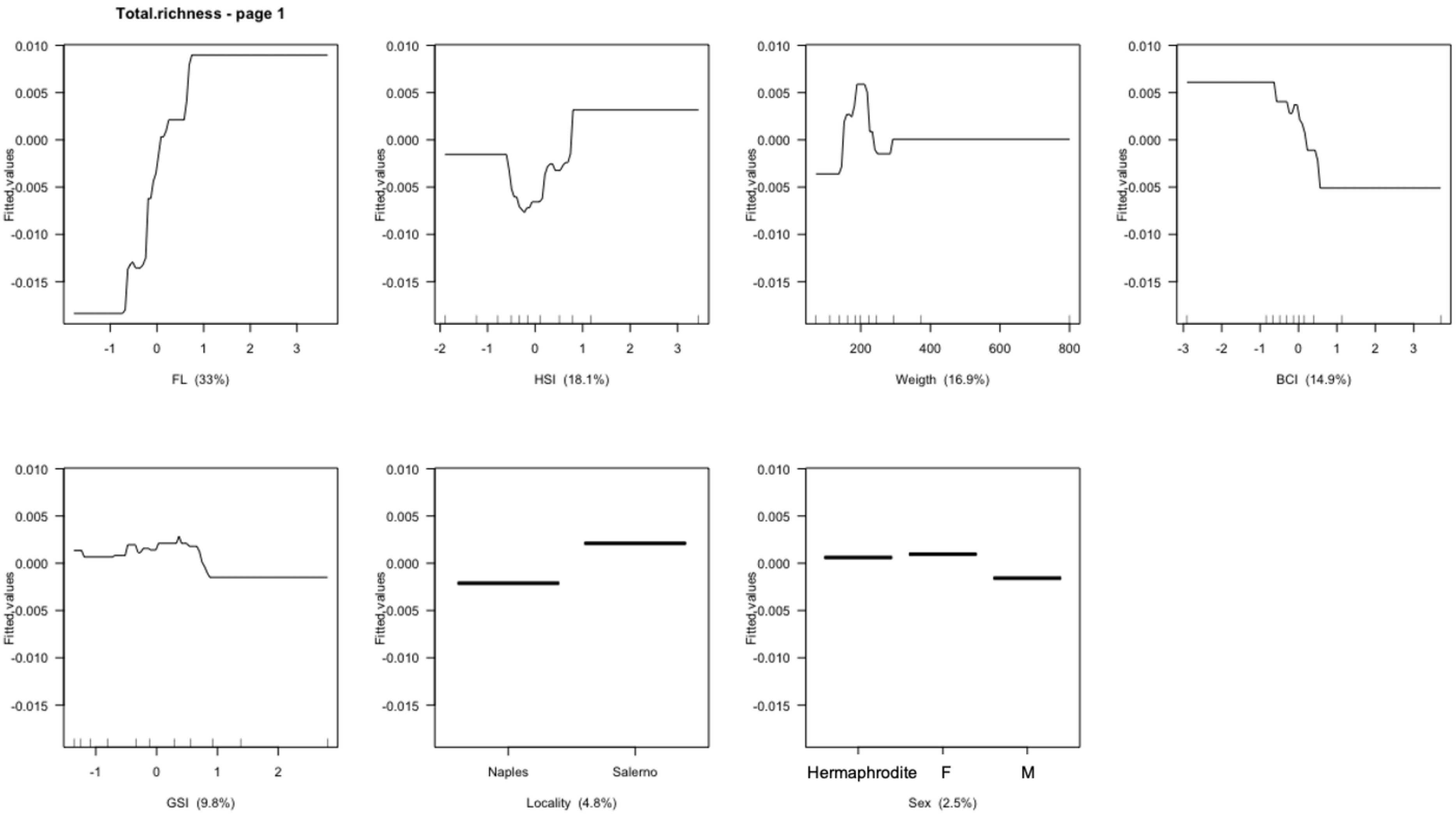
Total.richness - page 1
Fitted values
FL (33%)
HSI (18.1%)
Weigth (16.9%)
BCI (14.9%)
GSI (9.8%)
Naples
Salerno
Locality (4.8%)
Hermaphrodite
F
M
Sex (2.5%)

Figure S17 -

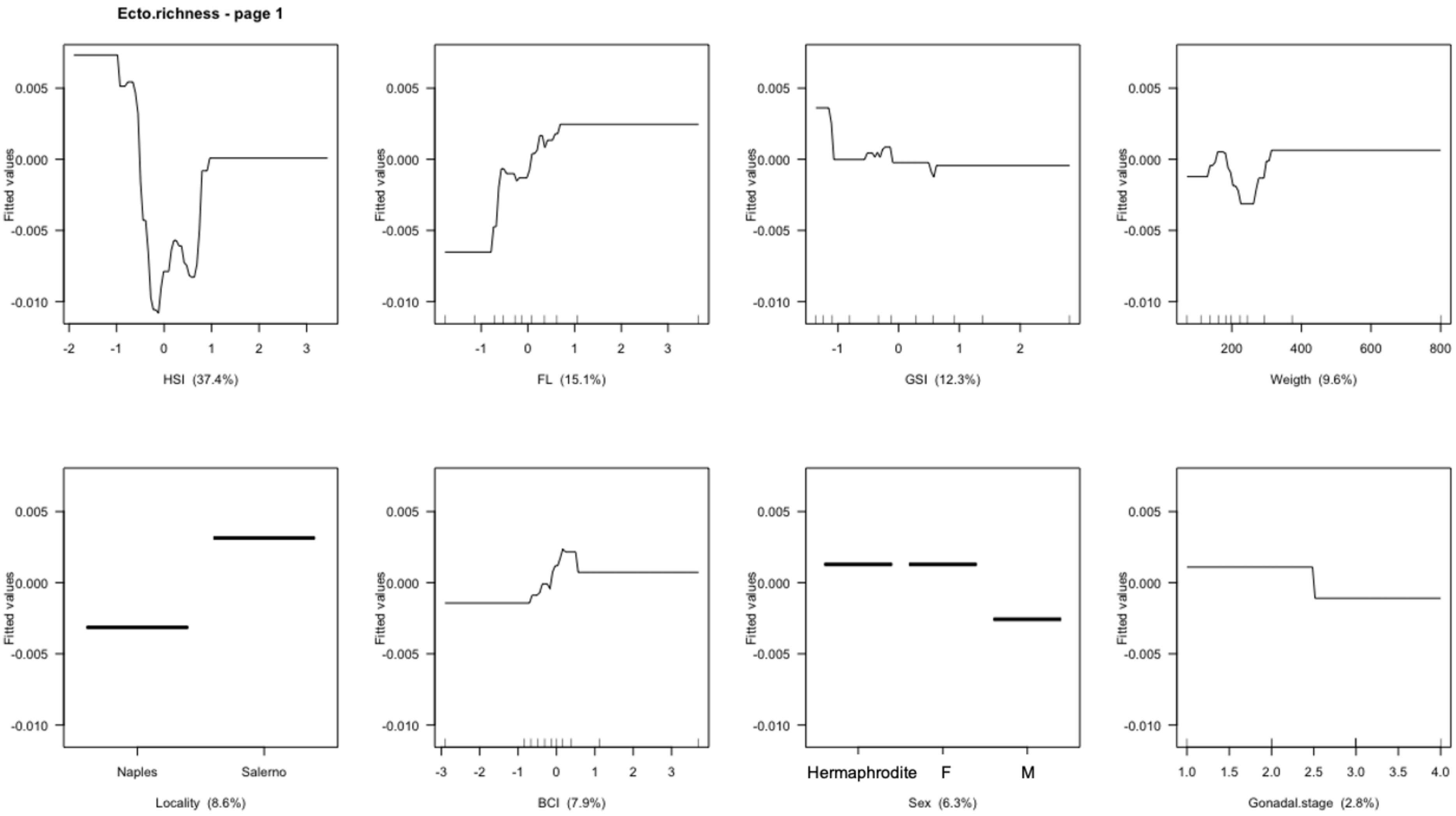
Ecto.richness - page 1
Fitted values
HSI (37.4%)
FL (15.1%)
GSI (12.3%)
Weigth (9.6%)
Naples
Salerno
Locality (8.6%)
BCI (7.9%)
Hermaphrodite
F
M
Sex (6.3%)
Gonadal.stage (2.8%)

Figure S18 -

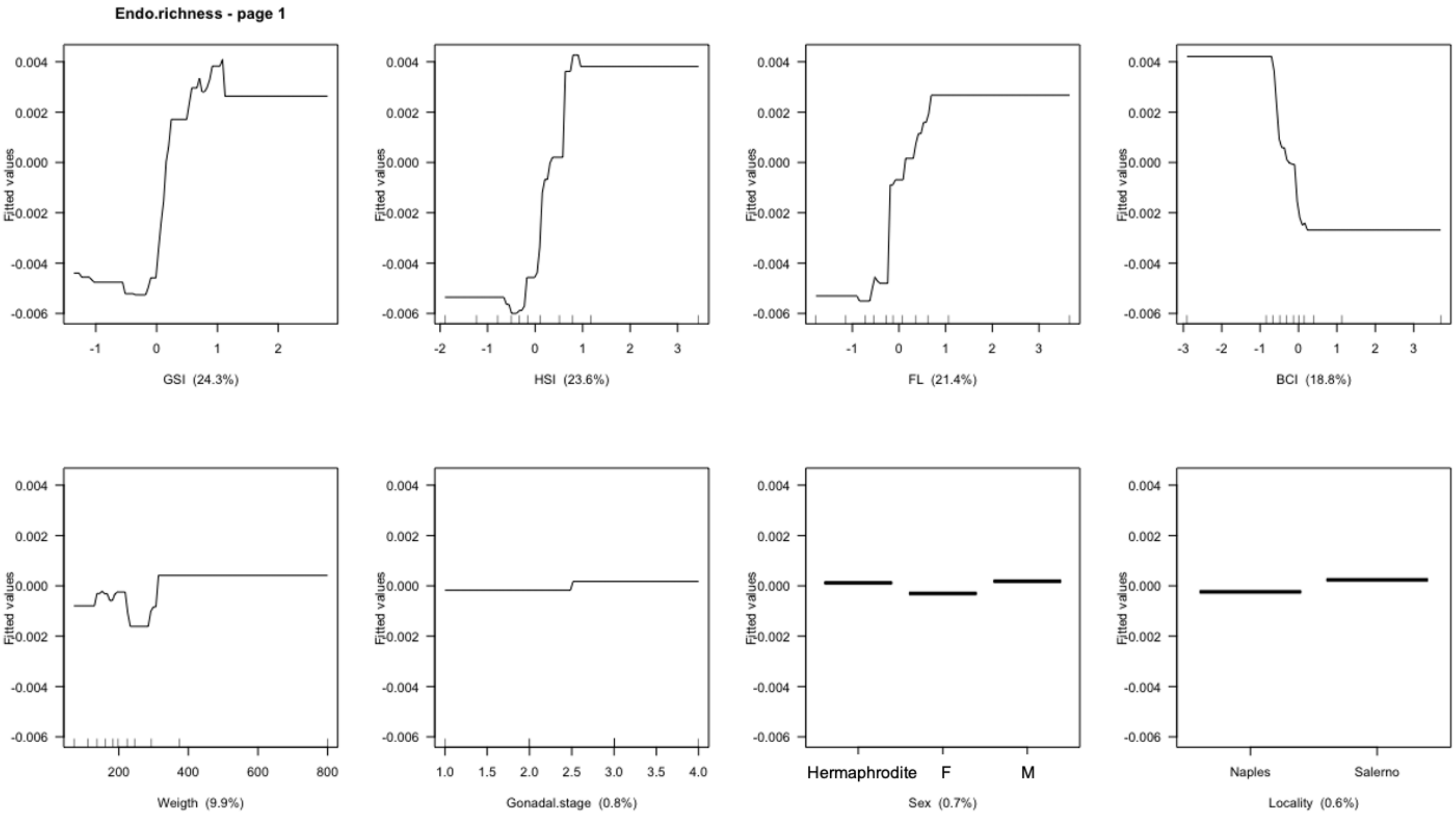

Endo.richness - page 1
Fitted values
GSI (24.3%)
HSI (23.6%)
FL (21.4%)
BCI (18.8%)
Weigth (9.9%)
Gonadal.stage (0.8%)
Hermaphrodite
F
M
Sex (0.7%)
Naples
Salerno
Locality (0.6%)

Figure S19 -

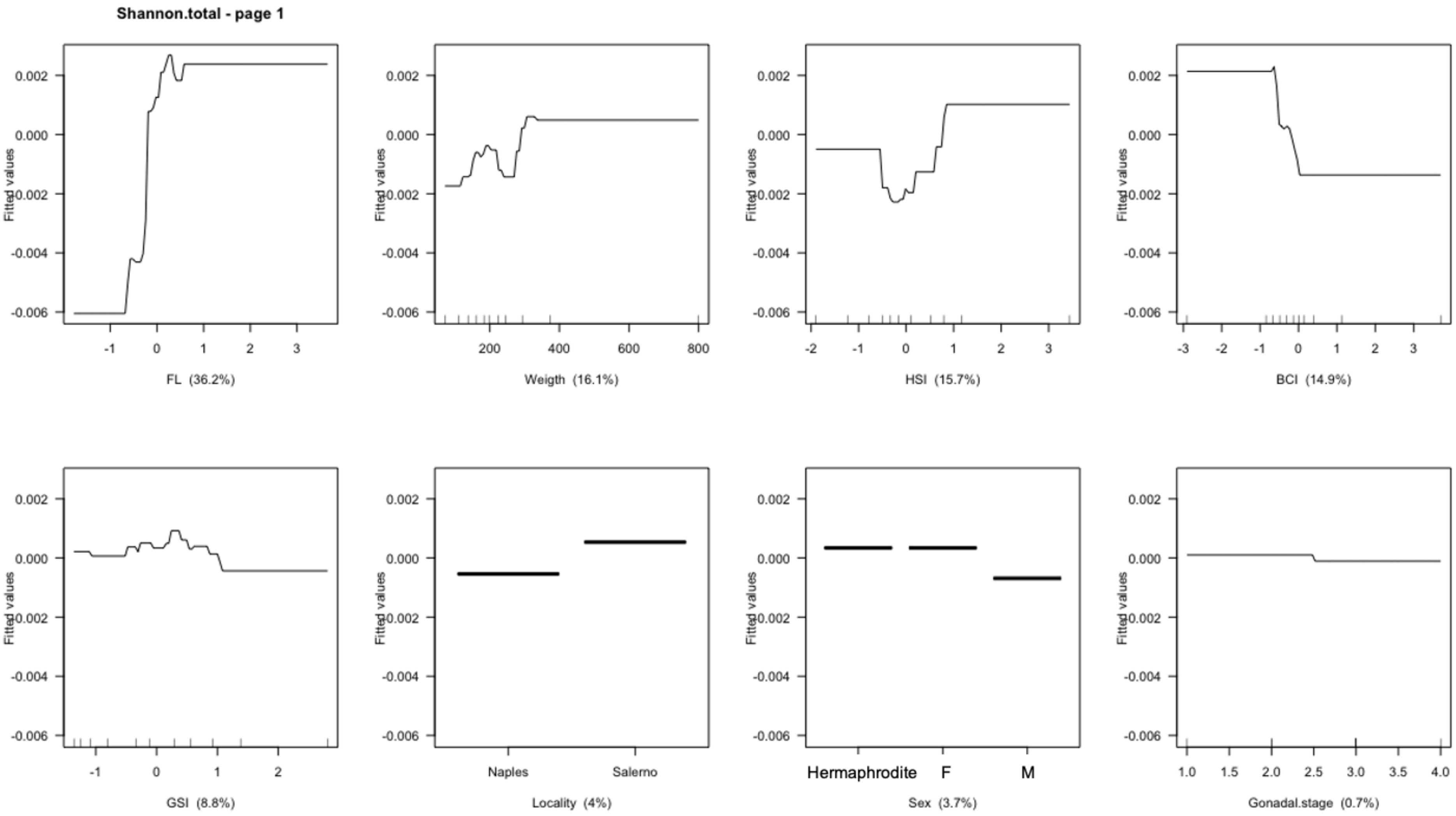
Shannon.total - page 1
Fitted values
FL (36.2%)
Weigth (16.1%)
HSI (15.7%)
BCI (14.9%)
GSI (8.8%)
Locality (4%)
Naples
Salerno
Sex (3.7%)
Hermaphrodite
F
M
Gonadal.stage (0.7%)

Figure S20 -

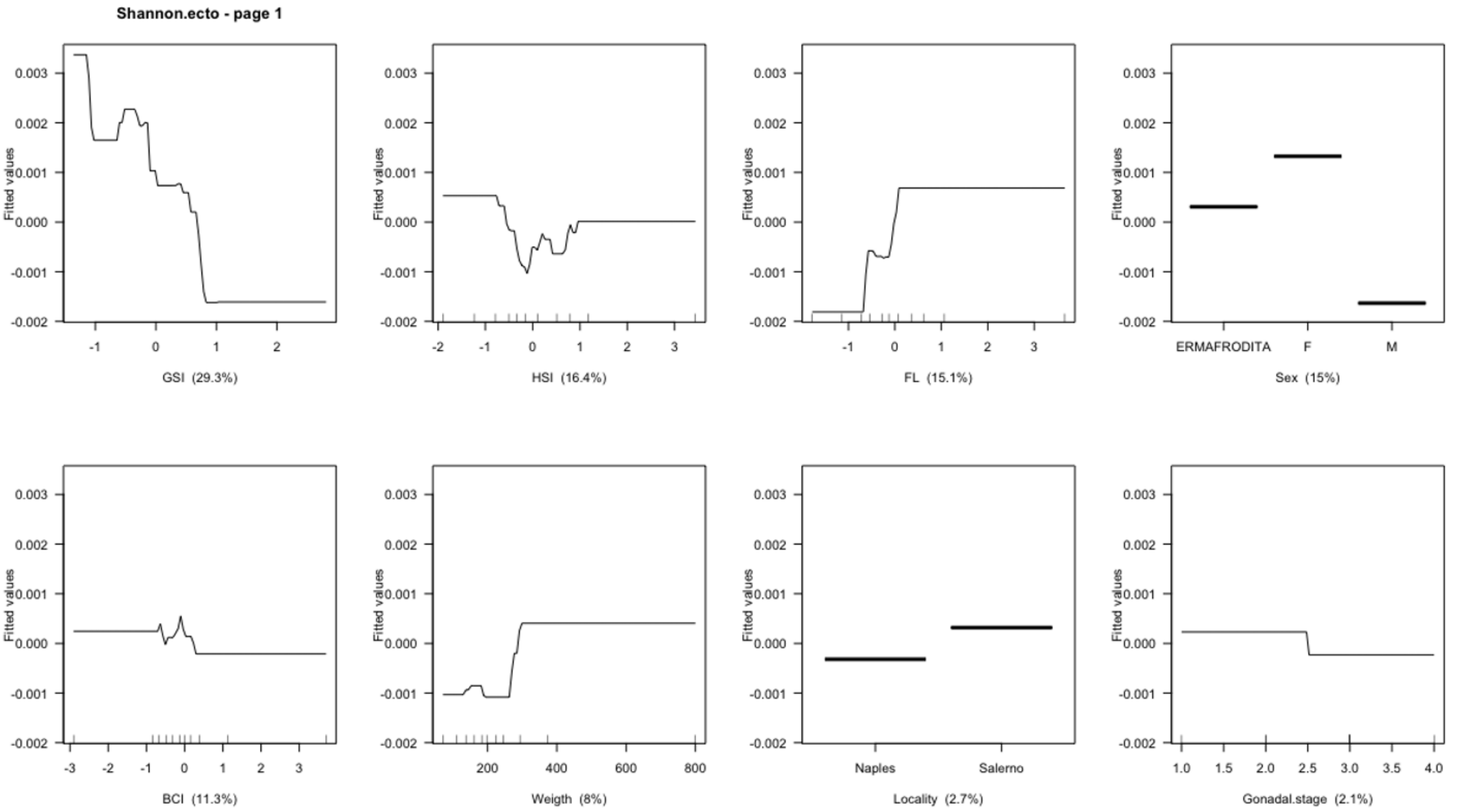

Shannon.ecto - page 1
Fitted values
GSI (29.3%)
HSI (16.4%)
FL (15.1%)
Sex (15%)
ERMAFRODITA
F
M
BCI (11.3%)
Weigth (8%)
Locality (2.7%)
Naples
Salerno
Gonadal.stage (2.1%)

Figure S21 -

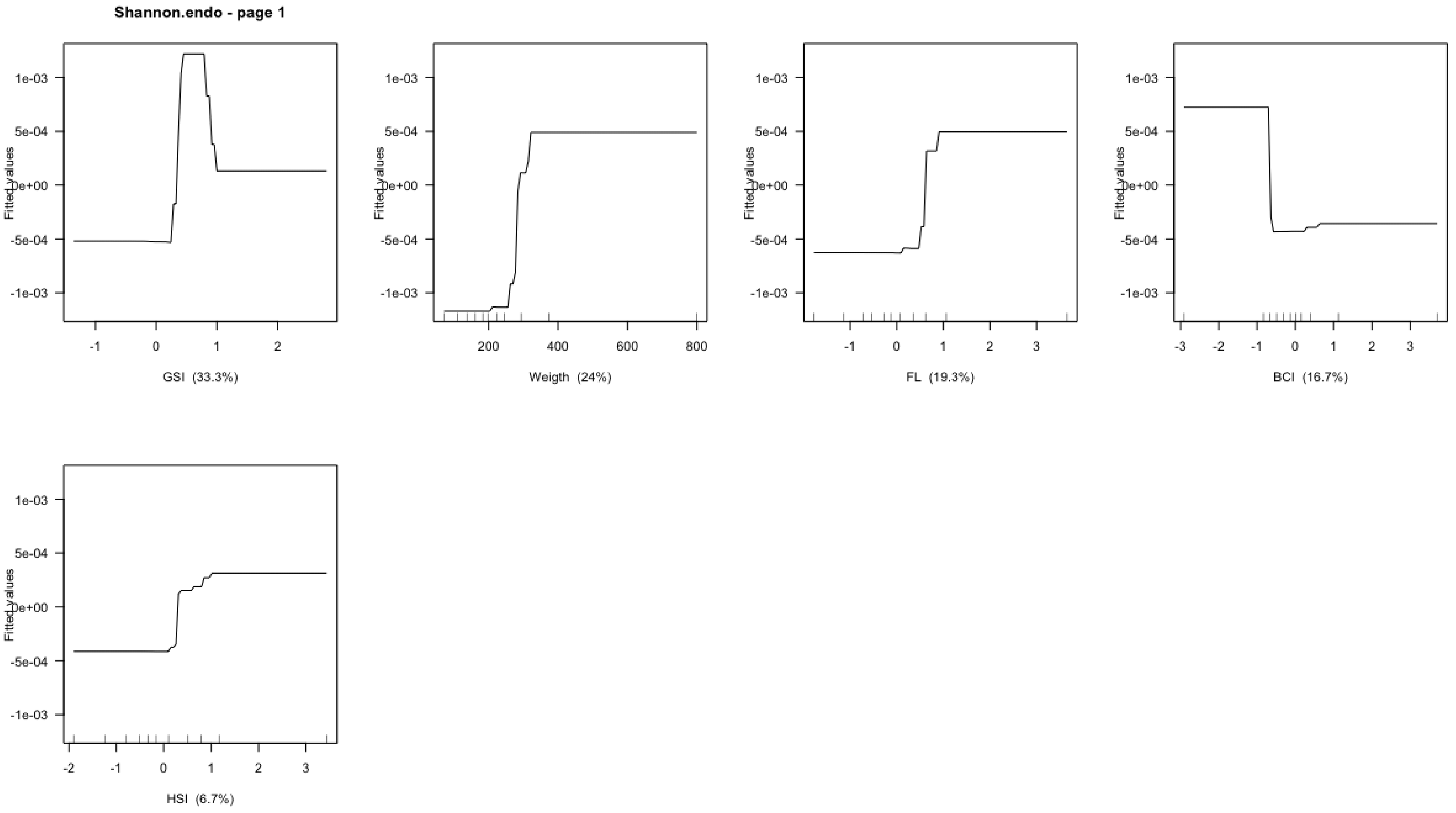
Shannon.endo - page 1
Fitted values
1e-03
5e-04
0e+00
-5e-04
-1e-03
GSI (33.3%)
Weigth (24%)
FL (19.3%)
BCI (16.7%)
HSI (6.7%)

Figure S22 -

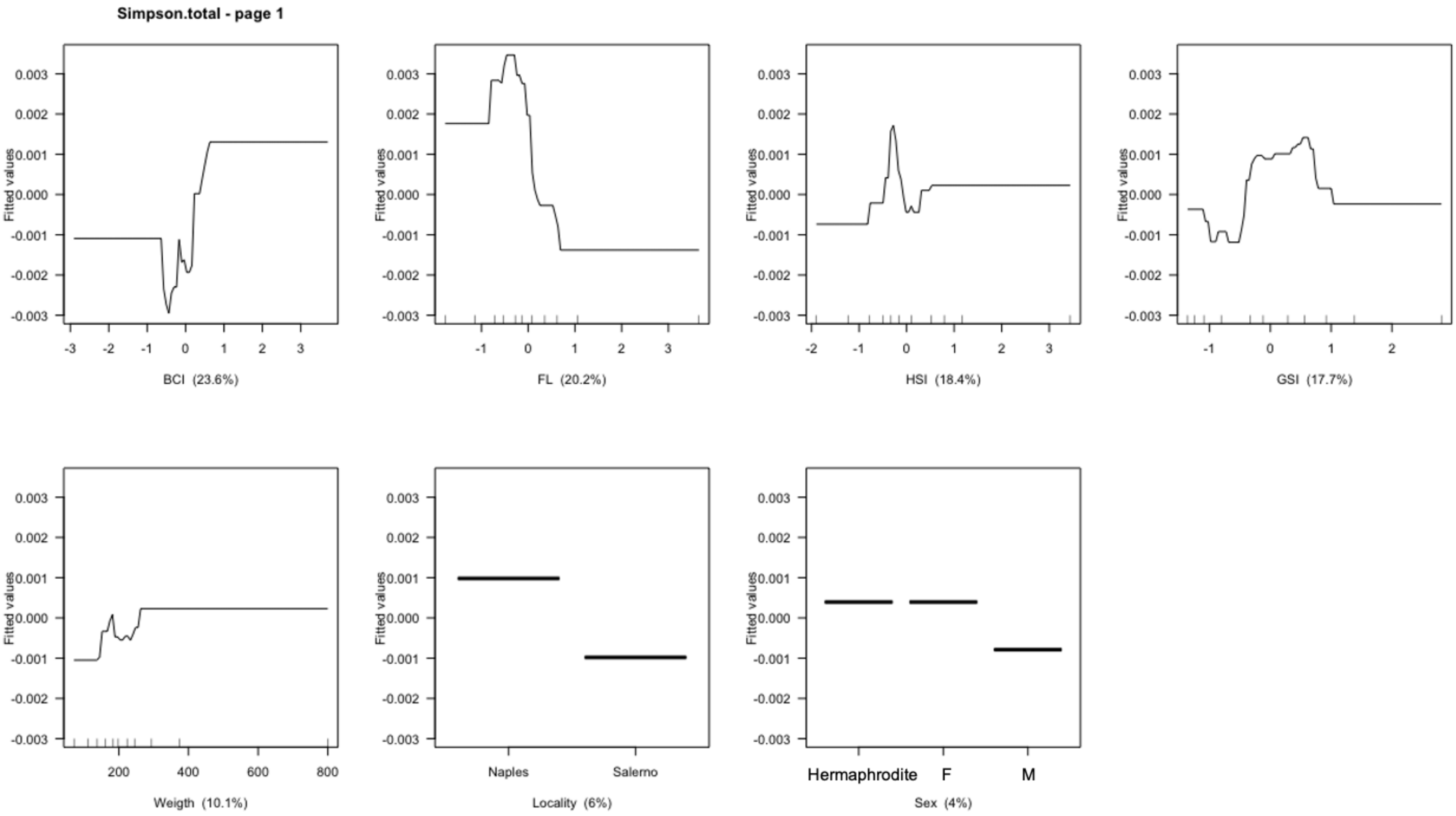
Simpson.total - page 1
Fitted values
BCI (23.6%)
FL (20.2%)
HSI (18.4%)
GSI (17.7%)
Weigth (10.1%)
Naples
Salerno
Locality (6%)
Hermaphrodite
F
M
Sex (4%)

Figure S23 -

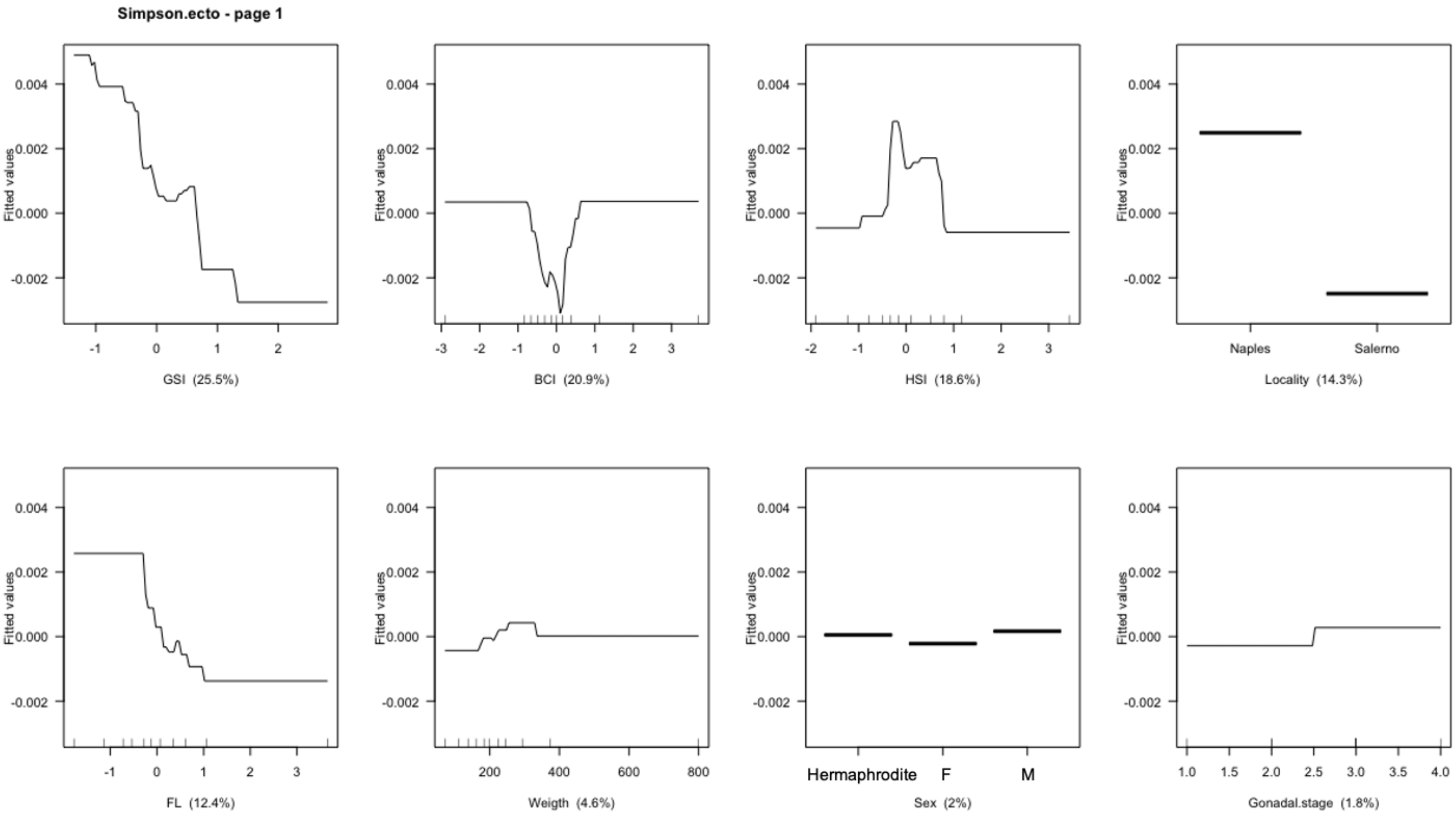
Simpson.ecto - page 1
Fitted values
0.004
0.002
0.000
-0.002
GSI (25.5%)
BCI (20.9%)
HSI (18.6%)
Naples
Salerno
Locality (14.3%)
FL (12.4%)
Weigth (4.6%)
Hermaphrodite
F
M
Sex (2%)
Gonadal.stage (1.8%)

Figure S24 -

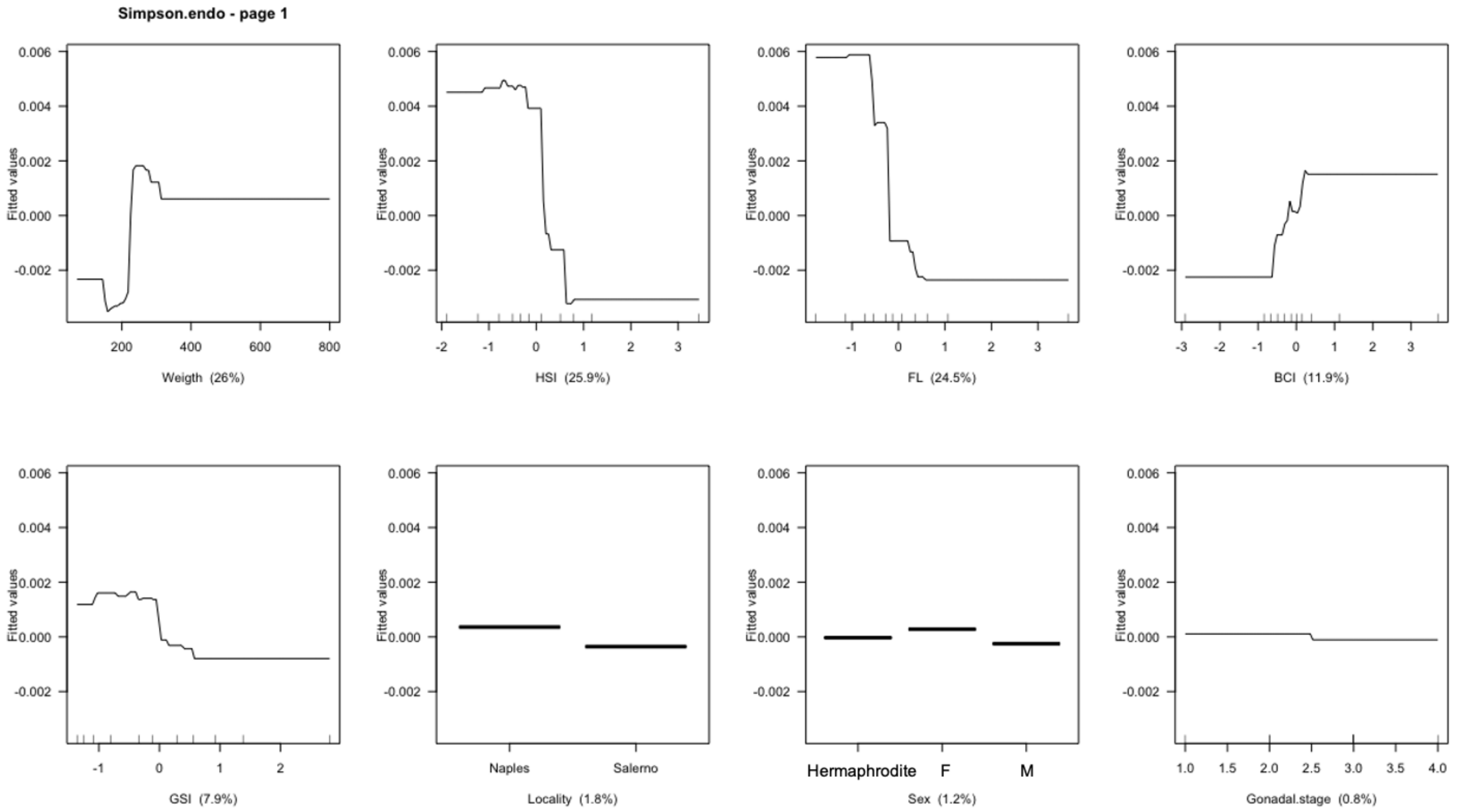
Simpson.endo - page 1
Fitted values
Weigth (26%)
HSI (25.9%)
FL (24.5%)
BCI (11.9%)
GSI (7.9%)
Naples
Salerno
Locality (1.8%)
Hermaphrodite
F
M
Sex (1.2%)
Gonadal.stage (0.8%)
